# Preservation of microvascular barrier function requires CD31 receptor-induced metabolic reprogramming

**DOI:** 10.1038/s41467-020-17329-8

**Published:** 2020-07-17

**Authors:** Kenneth C. P. Cheung, Silvia Fanti, Claudio Mauro, Guosu Wang, Anitha S. Nair, Hongmei Fu, Silvia Angeletti, Silvia Spoto, Marta Fogolari, Francesco Romano, Dunja Aksentijevic, Weiwei Liu, Baiying Li, Lixin Cheng, Liwen Jiang, Juho Vuononvirta, Thanushiyan R. Poobalasingam, David M. Smith, Massimo Ciccozzi, Egle Solito, Federica M. Marelli-Berg

**Affiliations:** 1 William Harvey Research Institute, Barts and The London School of Medicine and Dentistry, Queen Mary University of London, Charterhouse Square, London, EC1M 6BQ UK; 2 School of Life Sciences, Centre for Cell & Developmental Biology and Partner State Key Laboratory of Agrobiotechnology, The Chinese University of Hong Kong, Hong Kong SAR, China; 3 Institute of Inflammation and Ageing, College of Medical and Dental Sciences, University of Birmingham, Mindelson Way, Birmingham, B152WB UK; 4 Unit of Clinical Laboratory Science, University Campus Bio-Medico of Rome, Rome, Italy; 5 Internal Medicine Department, University campus Bio-Medico of Rome, Rome, Italy; 6 School of Biological and Chemical Sciences, Queen Mary University of London, Mile End Road, London, E1 4NS UK; 7 Department of Head and Neck Surgery, Sun Yat-sen University Cancer Center, State Key Laboratory of Oncology in South China, Guangzhou, 510060 People’s Republic of China; 8 AstraZeneca R&D, Cambridge Science Park, Milton Road, Cambridge, CB4 0WG UK; 9 Unit of Medical Statistic and Molecular Epidemiology, University Campus Bio-Medico of Rome, Rome, Italy; 10 Dipartimento di Medicina Molecolare e Biotecnologie Mediche, Universita degli studi di Napoli “Federico II”, 80131 Naples, Italy; 11 Centre for inflammation and Therapeutic Innovation, Queen Mary University of London, Charterhouse Square, London, UK

**Keywords:** Cell biology, Cell migration, Inflammation

## Abstract

Endothelial barrier (EB) breaching is a frequent event during inflammation, and it is followed by the rapid recovery of microvascular integrity. The molecular mechanisms of EB recovery are poorly understood. Triggering of MHC molecules by migrating T-cells is a minimal signal capable of inducing endothelial contraction and transient microvascular leakage. Using this model, we show that EB recovery requires a CD31 receptor-induced, robust glycolytic response sustaining junction re-annealing. Mechanistically, this response involves src-homology phosphatase activation leading to Akt-mediated nuclear exclusion of FoxO1 and concomitant β-catenin translocation to the nucleus, collectively leading to *cMyc* transcription. CD31 signals also sustain mitochondrial respiration, however this pathway does not contribute to junction remodeling. We further show that pathologic microvascular leakage in CD31-deficient mice can be corrected by enhancing the glycolytic flux via pharmacological Akt or AMPK activation, thus providing a molecular platform for the therapeutic control of EB response.

## Abstract (web summary)

The mechanisms that restore endothelial barrier integrity following inflammation-induced breaching are incompletely understood. Here the authors show that the CD31 immune receptor contributes to reestablishing vascular integrity via its effects on endothelial cell metabolism.

## Introduction

Physiological hyper-permeability is a typical microvasculature inflammatory response of, which facilitates diffusion of essential blood-borne immunoregulatory and pro-inflammatory mediators into extravascular tissue. Microvascular leakage results from a tightly regulated process induced by a wide range of inflammatory mediators. This event leads to phosphorylation and endocytosis of junctional vascular endothelial cadherin (VE-cadherin) complex and EC actomyosin contractility^1,2^. Endothelial contraction can open junctions via a number of molecular mediators^1,2^. Microvascular leakage can be accompanied by leukocyte extravasation^3,4^. For example, we have shown that major histocompatibility complex (MHC) molecule-ligation by migrating T lymphocytes or antibodies induces transient microvascular leakage to facilitate T-cell extravasation^5^. Mechanistically, EC MHC-class-I engagement induces a rapid translocation of RhoA to the cell membrane associated with F-actin stress fiber formation and cytoskeleton reorganization leading to cell contraction^6^. This effect is physiologically relevant to human disease, as capillary-leak syndrome is a key feature of vascularized allografts rejection^7–9^.

Following leukocyte crossing, microvascular integrity quickly recovers through re-annealing of inter-endothelial junctions, but the molecular basis of this process is poorly defined. Most mechanistic studies have been carried out in-vitro^10^. In-vivo studies have used EC-contraction mediators such as thrombin and LPS which also affect other cellular functions and cells, particularly immune cells^11,12^ thus adding confounding factors which prevent the mechanistic study of EBF recovery.

Inter-endothelial CD31-homophilic interactions are required to recover vascular integrity following MHC-stimulation by migrating T-lymphocytes^5^. CD31 is a member of the immunoglobulin gene superfamily expressed at high density at the lateral borders of endothelial cells^13^. CD31 cytoplasmic tail contains two immunoreceptor-tyrosine-based-inhibitory-motifs (ITIM), which upon activation, specifically recruit src-homology 2 tyrosine phosphatases SHP-2 and SHP-1. Although CD31-deficient mice do not spontaneously develop vascular leakage at steady state, CD31-deficiency has been associated with excessive vascular leakage following endothelial contraction induced by histamine^14^, thrombin^15^, and LPS-induced endotoxemia^16,17^. Importantly, loss CD31 expression by EC leads to uncontrolled T-cell extravasation in inflammatory conditions^18,19^. While this evidence points to a non-redundant role of CD31 in the recovery of endothelial continuity after barrier breach by stimuli inducing endothelial contraction, the molecular mechanisms are to-date unclear.

We have investigated the molecular mechanism of EBF recovery in response to endothelial contraction and junction release-inducing stimuli using in-vitro and in-vivo models of MHC-induced vascular leakage in the context of inter-endothelial CD31 interactions. We show that the re-establishment of microvascular integrity is dependent on a CD31-induced glycolytic response, which sustains coordinated cytoskeletal remodeling and junctional reassembly.

## Results

### CD31 is required for endothelial barrier recovery

To investigate the molecular mechanisms involved in the recovery of endothelial barrier function (EBF) we conducted preliminary studies to monitor the kinetics of endothelial permeability induced by MHC-class-I molecule signals (via antibody-ligation) in the presence or absence of CD31 co-engagement in confluent, WT and *cd31*^−/−^ EC monolayers. Treatment with IFN-γ was used to enhance MHC and ICAM-1 molecule expression, which is low in cultured endothelium^20^, as we have previously described^5^. As expected, antibody-triggering of MHC led to a quick and similar reduction in trans endothelial electrical resistance (TEER) by both WT and *cd31*^*−/−*^ endothelium (Supplementary Fig. 1a). However, while permeability of WT endothelium returned to baseline levels within 6 h, resistance of CD31-deficient endothelium remained significantly lower for up to 24 h after MHC-stimulation. TEER was not significantly affected by ICAM-1 Ab-mediated stimulation in conditions capable to induce Erk phosphorylation either in the presence or absence of CD31 expression, (Supplementary Fig. 1b). MHC-triggering did not induce EC death (Supplementary Fig. 1c).

**Fig. 1:**
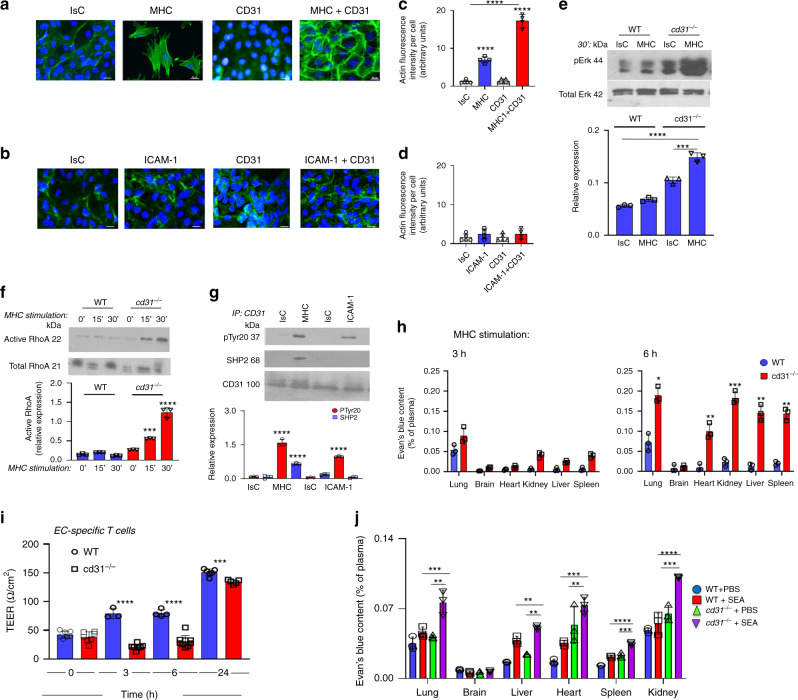
CD31 interactions promote the recovery of endothelial integrity following endothelial contraction induced by MHC molecule triggering. **a**–**d** Following MHC or ICAM-1 and/or CD31 antibody-mediated co-ligation for 30 min, EC were fixed and stained with rhodamine-phalloidin. Images taken on EC monolayers seeded at identical density are shown in (**a**, **b**). The average F-actin intensity per cell of three independent experiments is shown in (**c**, **d**). Scale bar, 20 μm. (*n* = 3 biologically independent samples, *N* = 3 independent experiments, data are mean ± SD). One-way Anova with Tuckey post-hoc test. MHC vs all ****p < 0.0001, MHC + CD31 vs Isc ****p < 0.0001, MHC + CD31 vs all ****p < 0.0001. **e** Western blot (WB) analysis of Erk activation by WT and *cd31*^*−/−*^ EC 30 min after MHC stimulation. The bar graph shows relative protein expression ± SEM. *N* = 3 independent experiments (data are mean ± SD). One-way Anova with Tuckey post-hoc test. *cd31*^*−/−*^ MHC vs *cd31*^*−/−*^ IsC ***p = 0.0002, *cd31*^*−/−*^ MHC vs all ****p < 0.0001. **f** Western blot (WB) analysis of RhoA activation by WT and *cd31*^*−/−*^ EC 30 min after MHC stimulation. The bar graph shows relative protein expression ± SEM. *N* = 3 independent experiments (data are mean ± SD). One-way Anova with Tuckey post-hoc test. *cd31*^*−/−*^ 15′ vs all ***p = 0.0003, *cd31*^*−/−*^ 30′ vs all ****p < 0.0001. **g** Immunoprecipitation of CD31 molecules from WT EC exposed to MHC/ICAM-1 stimulation for 30 min followed by immunoblotting with an anti-phosphotyrosine antibody and an anti-SHP2 antibody. The bar graph shows relative protein expression ± SEM. *N* = 3 independent experiments. One-way Anova with Tuckey post-hoc test. pTyr20 MHC vs all ****p < 0.0001, SHP2 MHC vs all ****p < 0.0001, pTyr20 ICAM-1 vs all ****p < 0.0001. **h** WT or *cd31*^*−/−*^ mice (*n* = 6 mice, *N* = 2 independent experiments) received anti-MHC and secondary cross-linking antibody (0.67 μg and 0.33 μg/kg body weight, respectively) in saline solution i.v. After 3 or 6 h, 100 μL of 2% Evans blue in saline solution was injected. Dye was allowed to circulate for 45 min before organs dye content was assessed spectrophotometrically and normalized to plasma levels (data are mean ± SEM). One-way Anova with Tuckey post-hoc test. Lung *cd31*^*−/−*^ vs WT *p = 0.0352, heart *cd31*^*−/−*^ vs WT **p = 0.0045, kidney *cd31*^*−/−*^ vs WT ***p = 0.0009, liver *cd31*^*−/−*^ vs WT **p = 0.0071, spleen *cd31*^*−/−*^ vs WT **p = 0.002. **i** H-2B-specific alloreactive T-cells were obtained by stimulation of Balb/C mice-derived splenocytes (H2-D) with sublethally irradiated WT splenocytes in the presence of 20 U/ml IL-2. T-cells were harvested 72 h after stimulation and seeded (10^5^/well) onto confluent WT or *cd31*^*−/−*^ EC monolayers grown on 0.2 μm-pore transwells and previously treated with IFN-γ for 48 h to upregulate MHC expression. TEER was measured as described in the Methods section. *n* = 3 biologically independent samples, *N* = 2 independent experiments. (data are mean ± SD) One-way Anova with Tuckey post-hoc test. 3 h WT vs cd31^*−*/*−*^ ****p < 0.0001, 6 h WT vs cd31^*−*/*−*^ ****p < 0.0001, 24 h WT vs cd31^*−*/*−*^ ***p = 0.0005. **j** WT or *cd31*^*−/−*^ mice (*n* = 6 mice, *N* = 2 independent experiments) were treated either with saline solution or with IFN-γ i.p. (70,000 U/mouse) i.p. After 48 h, some mice received an i.v. injection of the SEA superantigen (60 ng/mouse). After a further 4 h, 100 μL of 2% Evans blue in saline solution was injected. Dye was allowed to circulate for 45 min before organs dye content was assessed spectrophotometrically and normalized to plasma levels (data are mean ± SEM). One-way Anova with Tuckey post-hoc test. lung cd31^*−*/*−*^ SEA vs WT SEA ***p = 0.0006, lung cd31^*−*/*−*^ SEA vs cd31^*−*/*−*^ PBS **p = 0.002, liver cd31^*−*/*−*^ SEA vs WT SEA **p = 0.00124, liver cd31^*−*/*−*^ SEA vs cd31^*−*/*−*^ PBS ** = 0.004, heart cd31^*−*/*−*^ SEA vs WT SEA ***p = 0.0007, heart cd31^*−*/*−*^ SEA vs cd31^*−*/*−*^ PBS **p = 0.0088, spleen cd31^*−*/*−*^ SEA vs WT SEA ****p < 0.0001, spleen cd31^*−*/*−*^ SEA vs cd31^*−*/*−*^ ***p = 0.0002, kidney spleen cd31^*−*/*−*^ SEA vs WT SEA ****p < 0.0001, kidney cd31^*−*/*−*^ SEA vs cd31^*−*/*−*^ ***p = 0.0008.

As endothelial contractility is associated with F-actin polymerization and stress fiber formation^21^, we further analyzed EC cytoskeletal rearrangements following MHC-triggering with or without CD31 ligation. Sub-confluent EC monolayers were used in these experiments to allow assessing the contribution of CD31 signals ‘in isolation’, i.e. via antibody activation, on actin polymerization. MHC-ligation led to the formation of polarized bundles of F-actin stress fibers and further separation from adjacent EC (Fig. 1a, b), a feature of EC contraction. The sparsity of the MHC-stimulated EC on the image is likely to reflect the strength of cell contraction, possibly even leading to cell detachment, as the EC were seeded in equal numbers. This was confirmed by experiments showing a similar actin configuration in MHC-stimulated CD31-deficient EC (Supplementary Fig. 1d), while CD31 triggering on its own did not elicit any effect, co-ligation with MHC molecules significantly increased F-actin polymerization above the levels induced by MHC-signals, which was however associated with the cortical actin cytoskeleton and accompanied by intercellular attachment, suggesting that CD31 is required for efficient anchorage of actin fibers to the intercellular junctions during EC contraction. ICAM-1 ligation induced a slight increase in actin polymerization without endothelial contraction (Fig. 1c, d) and was not affected by co-delivery of CD31-mediated signals. Experiments designed to define the molecular mechanisms of sustained permeability of MHC-stimulated *cd31*^*−/−*^ endothelium revealed enhanced Erk and RhoA small GTPase activation in CD31-deficient but not WT EC (Fig. 1e, f). This pathway has been previously shown to induce endothelial contraction^22^. Immunoprecipitation studies in confluent EC confirmed that CD31 becomes phosphorylated upon MHC- but not ICAM-1 molecule stimulation, and this leads to the recruitment of the Src Homology Phosphatase 2 (SHP2), a key mediator of CD31 signals (Fig. 1g).

To validate the functional consequences of these observations, we tested the effect of signals on vascular barrier function in-vivo. First, we used a selective minimal signal—i.e. MHC-antibody ligation—to avoid confounding effects related to the engagement of other endothelial receptors by T-cells. In addition, systemic administration of the antibody allowed comparing EBFin multiple organs, and estimate the specific effect of MHC-signals in the absence of other inflammatory signals. In these experiments IFN-γ was not administered to avoid confounding effects. A non-complement fixing anti-MHC-class-I antibody was mixed with a secondary antibody and injected intravenously in WT and CD31-deficient mice. An anti-ICAM-1 antibody plus specific secondary antibody was also administered as a control. After 3 and 6 h, mice were injected with Evans Blue i.v. and tracer extravasation was measured. Vascular permeability was significantly increased after 6 h in the lung, liver and kidney of *cd31*^*−/−*^, but not WT animals, which received anti-MHC antibody (Fig. 1h). Interestingly, no increase in the permeability of the brain vasculature was observed in either WT or *cd31*^*−/−*^ mice, likely due to low MHC expression typical of brain endothelium^23,24^. No effects were observed following ICAM1 stimulation (Supplementary Fig. 1e). Systemic MHC-stimulation elicited a vascular leakage similar to that of histamine (Supplementary Fig. 1f), a stimulus well known to induce EC contraction with amplified severity in *cd31*^*−/−*^ mice^17^.

We subsequently assessed the effect of MHC-triggering by T-cells both in-vitro and in-vivo.

In-vitro, allogeneic (H2-D) T-cells were generated by in-vitro stimulation with DC obtained from WT donors (H2-B) and subsequently seeded on IFN-γ-treated (48 h) WT or *cd31*^*−/−*^ EC monolayers grown on 0.2 μm pore transwells. TEER increased only in WT EC monolayers for the first 6 h, while it remained low in the *cd31*^*−/−*^ ECs (Fig. 1i). The absence of TEER decrease in this system compared to direct MHC-ligation is that adhering T-cells also contribute to higher TEER. In-vivo, we induced systemic T-cell:MHC cognate interactions by i.v. injection of staphylococcal enterotoxin A (SEA). After i.p. injection of IFN-γ 48 h earlier, SEA administration induced greater vascular leakage in CD31-deficient mice in all organs analyzed except the brain (Fig. 1j). IFN-γ alone also increased vascular permeability in some organs, but this was minimal compared to SEA administration (Supplementary Fig. 1g).

Integrity of intercellular junctions is a major determinant of permeability of the endothelium, and de-phosphorylation of VE-cadherin-catenin complexes in adherens junctions mediate both anchorage and mechanical coupling of the cytoskeleton of adjacent EC including during leukocyte extravasation^1,2,25,26^.

**Fig. 2:**
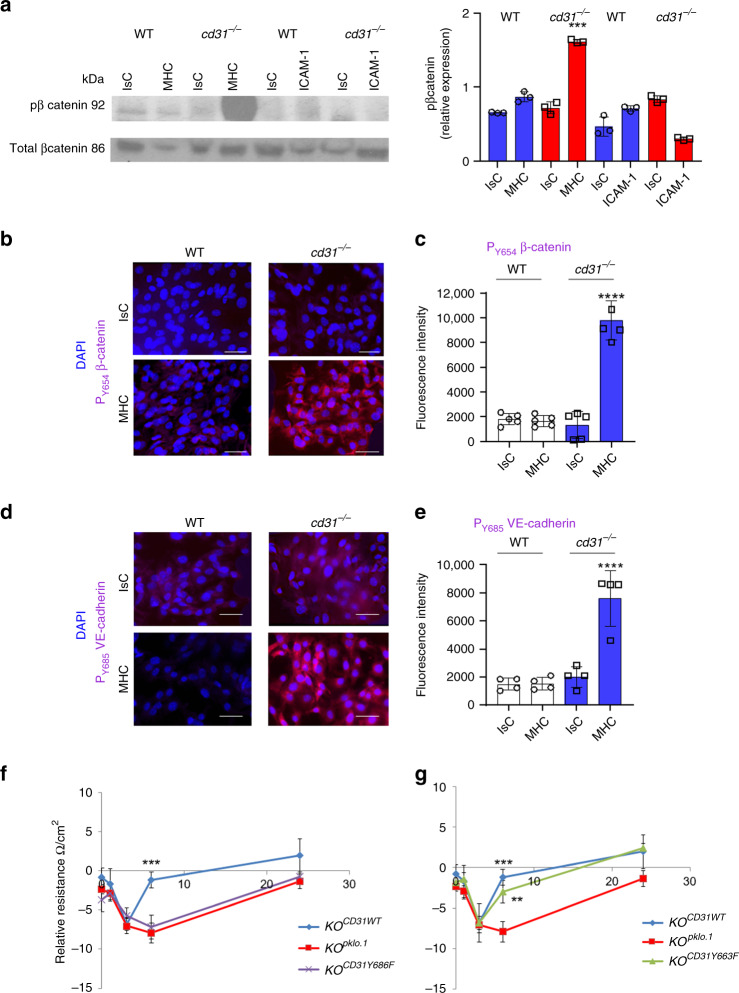
CD31 signals prevent VE-cadherin and β-catenin phosphorylation in response to MHC stimulation via ITIM 686-thyrosine phosphorylation. **a** phosphorylation of β-catenin (Y654) by MHC- or ICAM-1-stimulated (30 min) WT or *cd31*^*−/−*^ EC was analyzed by western blotting. The bar graph shows relative protein expression ± SEM. (*N* = 3 independent experiments). One-way Anova with Tuckey post-hoc test. *cd31*^*−/−*^ MHC vs all ***p = 0.0002. **b**–**e** phosphorylation of β-catenin (Y654, **b**) or VE-cadherin (Y685, **d**) by MHC-stimulated (30 min) WT or CD31^*−*/*−*^ EC was analyzed by widefield fluorescence microscopy. The bar graphs **c**, **e** show the mean fluorescence intensity/per cell of the indicated marker measured in three independent experiments by ImageJ software. Scale bar, 20 μm. Magnification ×20. Data are mean ± SD. One-way Anova with Tuckey post-hoc test. **c** cd31^*−*/*−*^ MHC vs all ****p < 0.0001; **e** cd31^*−*/*−*^ MHC vs all ****p < 0.0001. **f**, **g** CD31 gene constructs with mutations leading to the loss-of-function amino acid substitutions Y663F and Y686F in the ITIMs were generated and expressed by lentiviral transduction into *cd31*^*−/−*^ (KO) ECs (*KO*^*CD31Y686F*^*, KO*^*CD31Y663F*^). As a control, CD31 KO ECs were transduced with a wild-type CD31 gene construct (*KO*^*CD31WT*^) or an empty plasmid (*KO*^*pklo.1*^). ECs (6 × 10^4^/well) previously treated with 300 U/ml IFN-γ for 48 h (to enhance MHC molecule and ICAM-1 expression) were seeded onto 0.2 μm-pore transwells and stimulated with 5 μg/ml anti-mouse H-2Ld/H-2Db, or relevant isotype control followed by a secondary cross-linking Ab. TEER was measured as described in the Methods section. *n* = 3 biologically independent samples, *N* = 2 independent experiments. Data are mean ± SD. One-way Anova with Tuckey post-hoc test. **f** KO ^CD31WT^ vs all ***p  = 0.0002; **g** KO ^CD31WT^ vs all ***p = 0.0002, KO^Y663^ vs. KO ^pklo.1^ **p = 0.0024.

We therefore analyzed β-catenin phosphorylation in response to MHC- and ICAM-1-mediated signals by CD31-expressing or -deficient EC. Both MHC- and ICAM-1-ligation led to a modest increase in β-catenin phosphorylation at tyrosine 654 (Fig. 2a), which was substantially increased in the absence of CD31-mediated interactions during MHC- but not ICAM-1-triggering, indicating that CD31 modulates MHC-induced β-catenin phosphorylation as confirmed by wide-field fluorescence microscopy **(**Fig. 2b, c**)**. Accordingly, VE-cadherin phosphorylation increased substantially in MHC-activated CD31-deficient EC (Fig. 2d, e).

The causative role of CD31-mediated signals on vascular integrity was confirmed by lentiviral transduction of *cd31*^*−/−*^ ECs with wild-type CD31-gene constructs (KO^CD31WT^) or an empty plasmid (KO^ploc.1^), or two CD31-gene constructs with mutation with loss-of-function amino acid substitutions Y663F and Y686F in the ITIMs (KO^CD31Y663F^, KO^CD31Y686F^ ECs) (Supplementary Fig. 2a, b). Reconstitution with an intact CD31 gene rescued the ability of *cd31*^*−/−*^ EC to recover barrier function upon MHC-ligation (Fig. 2f, g). This was also achieved by KO^CD31Y663F^ but not KO^CD31Y686F^ EC, suggesting that phosphorylation at amino-acid residue tyrosine 686, known to be essential for SHP2 recruitment by CD31 is instrumental for its ability to maintain barrier function^27^. Similar to *cd31*^*−/−*^, KO^CD31Y686F^ EC underwent VE-cadherin and β-catenin phosphorylation (Supplementary Fig. 2c, d). Conversely, tyrosine-663 but not -686 was instrumental for CD31 intracellular trafficking^28^, suggesting that the two ITIMs may differentially contribute to distinct CD31 functions. The role of CD31 was further confirmed in experiments in which CD31 and/or MHC were antibody-ligated on sparsely seeded WT EC which showed that Akt activation only occurred in the presence of CD31 ligation (Supplementary Fig. 2e, f), ruling out that MHC-mediated signals on their own induce this signaling pathway.

### CD31-induced metabolic reprogramming regulates EBF response

The observations above do not explain the intense membrane-associated cytoskeleton reorganization and EC contraction induced by MHC and CD31 co-engagement. Although Src-induced phosphorylation of VE-cadherin prevents the binding of β-catenin, this is not sufficient to increase endothelial permeability^29,30^.

EC metabolism has recently emerged as an important regulator of endothelial function^31–33^.

**Fig. 3:**
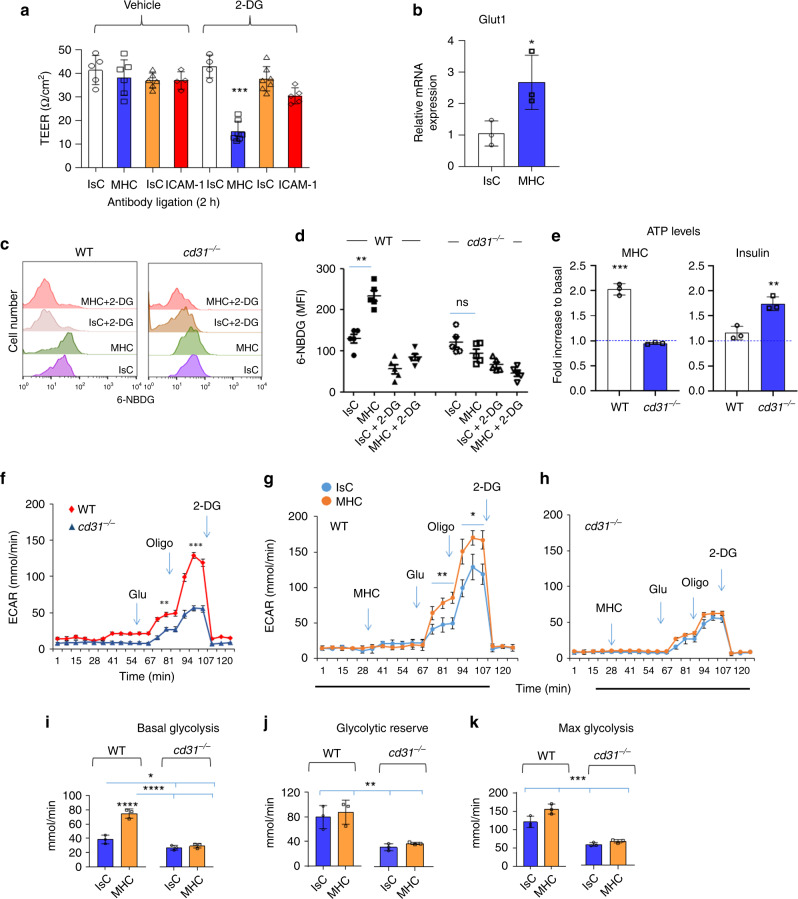
CD31 regulates EBF via its effects on EC metabolism. **a** EC were pre-treated with the glucose analog 2-DG (5 mM, 2 h) then MHC-1- or ICAM–stimulated for 4 h prior to TEER measurements. The bar graph shows the mean of measurement collected in three separate experiments of identical design ± SD. One-way Anova with Tuckey post-hoc test. 2DG MHC vs all ***p = 0.0003. **b** Glut-1 expression by EC following MHC stimulation (2 h) was evaluated by qRT-PCR. Data are mean ± SD. Two-tailed Student’s *T* test. MHC vs IsC *p = 0.0412. **c**, **d** Representative histograms of antibody-stimulated WT or CD31-deficient EC incubated with 6-NBDG for 2 h prior to analysis. 2-DG was used as a negative control. The graph shows the mean MFI measured in three independent experiments ± SD. One-way Anova with Tuckey post-hoc test. WT IsC vs WT MHC **p = 0.003, *cd31*^*−/−*^ IsC vs *cd31*^*−/−*^ MHC ns = 0.058. **e** ATP levels were measured in WT and CD31-deficient EC 4 h after MHC or insulin (1.8 µM) stimulation. Data are mean ± SD. Two-tailed Student’s *T* test. WT MHC vs *cd31*^*−/−*^ MHC ***p = 0.0002, WT insulin vs *cd31*^*−/−*^ insulin **p = 0.0062. Extracellular acidification rate (ECAR) of unstimulated and antibody-stimulated WT and CD31-deficient EC is shown in (**f**–**h**). The basal and maximal glycolysis and the glycolytic reserve are shown in (**i**–**k**). Wells were first injected with anti MHC antibodies at the indicated time point. Isotype-matched and secondary antibodies were used as controls. Further injections followed at the time point indicated (arrows) introducing the indicated compounds into the wells. *N* = 3 independent experiments. The error bars represent SD. One-way Anova with Tuckey post-hoc test. **f** WT time 81 vs *cd31*^*−/−*^ time 81 **p = 0.0023, WT time 94 vs *cd31*^*−/−*^ time 94 ***p = 0.0017; **h** IsC Glu/Oligo injection vs MHC Glu/Oligo **p = 0.012, IsC Oligo/2DG vs MHC Oligo/2DG *p = 0.027; **i** WT MHC vs WT IsC ****p < 0.0001, WT IsC vs *cd31*^*−/−*^ IsC/MHC *p = 0.0245, WT MHC vs *cd31*^*−/−*^ IsC/MHC ****p < 0.0001; **j** WT IsC vs *cd31*^*−/−*^ IsC **p = 0.0056, WT IsC vs *cd31*^*−/−*^ MHC **p = 0.0097, WT MHC vs *cd31*^*−/−*^ IsC **p = 0.0024, WT MHC vs *cd31*^*−/−*^ MHC **p = 0.0041; **k** WT IsC vs *cd31*^*−/−*^ IsC ***p = 0.0002, WT IsC vs *cd31*^*−/−*^ MHC ***p = 0.0003, WT MHC vs *cd31*^*−/−*^ IsC ***p = 0.00024, WT MHC vs *cd31*^*−/−*^ MHC ***p = 0.00021.

WT EC permeability following MHC-stimulation for 3 hours (i.e. when it would normally return to baseline) was dramatically increased by inhibition of glycolysis with the glucose analog 2-deoxyglucose (2-DG), indicating that recovery of junction stability upon MHC-triggering might depend on a robust glycolytic response (Fig. 3a).

We therefore investigated the effect of MHC- and CD31-mediated signals on EC metabolism. To avoid activation of metabolic pathways due to EC proliferation or migration these experiments were performed using confluent EC monolayers. Expression of the ubiquitous glucose transporter Glut1 and uptake of the glucose-analog 6-[N-7-nitrobemz-2-oxa-1,3-diazol-4-amino]−2-deoxyglucose (6-NBDG), which accumulates in the cytoplasm in its fluorescent form, were significantly increased in CD31-competent, but not -deficient EC following–stimulation (Fig. 3b–d, Supplementary Fig. 3a). Importantly, 2-DG pre-treatment of EC abrogated 6-NBDG uptake irrespective of CD31 expression, suggesting a positive feed-back loop of glycolysis itself on glucose uptake. We further observed that *cd31*^*−/−*^ EC failed to redistribute Glut1 upon MHC-stimulation, suggesting a defect in Glut1 recycling to the cell membrane (Supplementary Fig. 3b). Accordingly, ATP levels in response to MHC-triggering were significantly reduced in CD31-deficient EC (Fig. 3e). When stimulated with insulin, however, ATP levels were increased in CD31-deficient EC compared to WT EC. As insulin is a powerful inducer of glycolysis^34^, not supplemented in EC culture medium, CD31-deficient EC are capable to engage the glycolytic pathway in response to insulin, but not MHC-stimulation. Further, the possibility that addition of insulin in the insulin-free medium used might attenuate the effect of CD31-deficiency rather than engaging a CD31-independent pathway is ruled out by the in vivo experiments (i.e. in the presence of physiological amounts of insulin) which show that MHC stimulation induces vascular leakage in the absence of CD31 signals (see Fig. 1h).

To differentiate the effects of MHC-stimulation on aerobic glycolysis from other glucose-dependent functions (such as protein glycosylation), we measured lactate production by MHC-activated WT and *cd31*^*−/−*^ EC. The reduced glycolytic ability of *cd31*^*−/−*^ EC was confirmed by measurement of extracellular acidification rate (ECAR). The glycolytic flux in resting, confluent EC was significantly decreased in CD31-deficient EC, suggesting that inter-endothelial CD31 interactions support baseline glycolysis in resting endothelium (Fig. 3f). MHC-stimulation of WT, but not *cd31*^*−/−*^ EC, resulted in significantly enhanced ECAR (Fig. 3g, h). A glycolysis stress test confirmed that *cd31*^*−/−*^ EC display an impaired basal glycolytic response, reserve and maximal response **(**Fig. 3i–k**)**, independently of MHC-triggering. We further analyzed the effect of reconstitution of lentivirus-transduced *cd31*^*−/−*^ EC with either CD31^WT^, CD31^ploc.1^, CD31^Y663F^ and CD31^Y686F^ gene constructs on the glycolytic response to MHC-triggering and confirmed that tyrosine 686 is required for CD31 signals to elicit this metabolic response (Supplementary Fig. 3c)

**Fig. 4:**
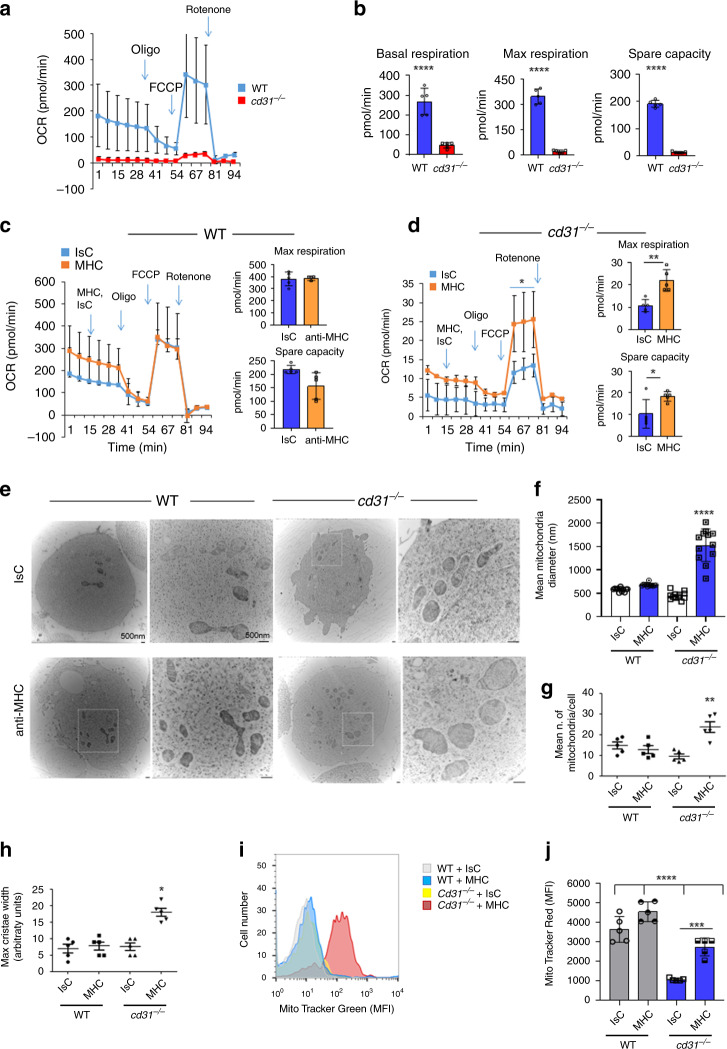
CD31-deficient EC increase mitochondrial respiration upon MHC stimulation. Oxygen consumption rate (OCR), basal, maximal respiration, and spare capacity of unstimulated WT and CD31-deficient EC is shown in (**a**, **b**), respectively (*N* = 3 independent experiments). The error bars represent SD. One-way Anova with Tuckey post-hoc test. Basal respiration WT vs *cd31*^*−/−*^ ****p < 0.0001, max respiration WT vs *cd31*^*−/−*^ ****p < 0.0001, spare capacity WT vs *cd31*^*−/−*^ ****p < 0.0001. Oxygen consumption rate (OCR) of unstimulated and MHC-stimulated WT and *cd31*^*−/−*^ EC is shown in (**c**, **d**), respectively. The maximal respiration and spare capacity are shown in the right-had-side of each panel. Wells were first injected with anti MHC antibodies at the indicated time point. Isotype matched and secondary antibodies were used as controls. Further injections followed at the time point indicated (arrows) introducing the indicated compounds into the wells. *N* = 3 independent experiments. The error bars represent SD. One-way Anova with Tuckey post-hoc test. **d** Rotenone injection IsC vs Rotenone injection MHC *p = 0.04, Max respiration IsC vs MHC **p = 0.002, spare capacity IsC vs MHC *p = 0.0327. **e** EM analysis of EC mitochondria and mitochondrial cristae of WT and *cd31*^*−/−*^ EC MHC- or IsC-stimulated over 4 h. Scale bar = 500 nm, represents 2 experiments. The mean transverse diameter and number of mitochondria measured in 15 images from each experimental group is shown in (**f**, **g**), respectively. In **h** maximum cristae width was measured from 15 images from each experimental group using ImageJ software and it is expressed in arbitrary units, Bar graphs depicts mean ± SEM (*N* = 3 independent experiments). One-way Anova with Tuckey post-hoc test. **f** *cd31*^*−/−*^ MHC vs all ****p < 0.0001; **g** *cd31*^*−/−*^ MHC vs all **p = 0.003; **h** *cd31*^*−/−*^ MHC vs all *p = 0.019. **i** MitoTracker Green staining of WT and *cd31*^*−/−*^ EC MHC- or IsC-stimulated for 4 h was analyzed by flow cytometry. Representative histograms from 2 replicate experiments are shown. **j** In similar experiments, MitoTracker Red fluorescence was measured from 15 images from each experimental group using ImageJ software, Bar graphs depicts mean ± SEM (*N* = 3 independent experiments). One-way Anova with Tuckey post-hoc test. WT IsC vs WT MHC ****p < 0.0001, WT IsC vs *cd31*^*−/−*^ IsC ****p < 0.0001, WT IsC vs *cd31*^*−/−*^ MHC ****p < 0.0001, WT MHC vs *cd31*^*−/−*^ IsC ****p < 0.0001, WT MHC vs *cd31*^*−/−*^ MHC ****p < 0.0001, *cd31*^*−/−*^ IsC vs cd31^*−*/*−*^ MHC ***p = 0.0002.

The oxygen consumption rate (OCR), a measure of mitochondrial respiration, was severely compromised in both resting and MHC-activated *cd31*^*−/−*^ EC (Fig. 4a, b). OCR was not affected by MHC-ligation in WT EC, while both maximal respiration and spare capacity were significantly enhanced in CD31-deficient EC (**c, d**), suggesting that in the absence of CD31 signals, EC engage mitochondrial respiration to meet energy demands. We therefore assessed mitochondria number, shape and cristae morphology MHC-activated WT and *cd31*^*−/−*^ EC. No differences in the number of mitochondria in resting EC were observed (Fig. 4e–g**)**. In contrast, MHC-stimulated, CD31-deficient EC displayed increased mitochondria size (**f**) and numbers (**g**), with increased cristae width compared to their WT counterpart (**h**). The overall increase in mitochondrial mass by MHC-stimulated *cd31*^*−/−*^ EC was confirmed by flow cytometry (**i**). Mitochondrial function this was found to be severely reduced in *cd31*^*−/−*^ EC (Fig. 4j), which nevertheless increased it in response to MHC-stimulation.

It has been shown that T-cells that preferentially utilize FAO, maintain fused mitochondria^35,36^. We, therefore, compared WT and CD31-deficient EC ability to oxidize glucose, glutamine and fatty acids (FAO). As shown in Supplementary Fig. 2a, glucose oxidation did not significantly differ in unstimulated WT and *cd31*^*−/−*^ EC. However, while glucose oxidation was increased in WT EC in response to MHC-signals, this pathway was impaired in CD31-deficient EC (Supplementary Fig. 3d). Glutamine oxidation, in contrast, was utilized more by resting CD31-deficient EC, which were however unable to increase it following MHC-stimulation (Supplementary Fig. 3e). FAO was significantly enhanced by MHC-stimulated CD31-deficient but not WT EC (Supplementary Fig. 3f). Accordingly, transcription of the mitochondrial enzyme carnitine palmitoyltransferase 1A (*cpt1a*), essential for FAO, was enhanced by MHC-stimulation in CD31-deficient endothelium (Supplementary Fig. 3g).

**Fig. 5:**
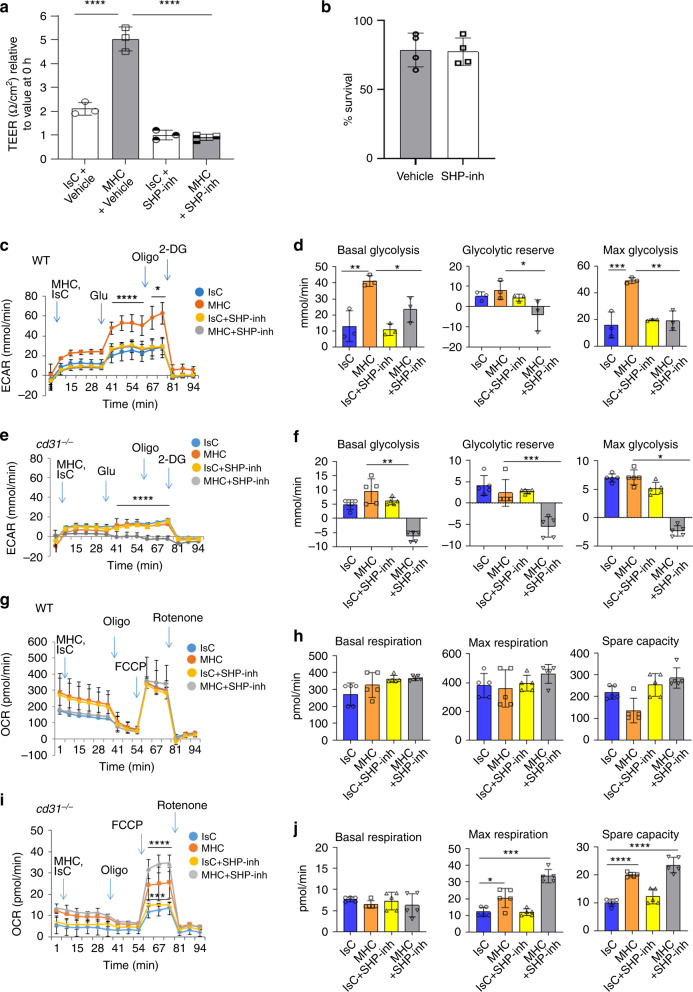
The metabolic response to endothelial barrier breach is src-phosphatase-dependent. Confluent EC monolayers were MHC-stimulated and treated with the selective SHP1/2 inhibitor (50 μM for 3 h) or vehicle as a control. **a** The bar graph shows the mean TEER values measured in three independent experiments. **b** Percentage of EC viability following treatment with SHP1/2 inhibitor determined by the trypan blue exclusion assay in three independent experiments. **c**–**f** ECAR measured in WT (**c**, **d**) or *cd31*^*−/−*^ EC (**e**, **f**) following MHC antibody-stimulation with or without pre-treatment with the SHP1/2 inhibitor (50μM for 45 min). Basal and maximal glycolysis and glycolytic reserve are shown in (**d**, **f**). **g**–**j** OCR measured in WT **g**–**h** or CD31-deficient EC **I**, **j** following MHC antibody-stimulation with or without pre-treatment with the SHP1/2 inhibitor. Basal and maximal respiration and spare capacity are shown in (**i**, **j**). (*N* = 3 independent experiments). Data are shown as mean ± SEM; significant differences were determined using one-way Anova with Tuckey post-hoc test. **a** IsC vehicle vs MHC vehicle ****p < 0.0001, MHC vehicle vs MHC + SHP inhibitor ****p < 0.0001; **c** Glu/Oligo injection WT MHC vs all ****p < 0.0001, Oligo/2DG injection WT MHC vs all *p = 0.0103; **d** basal glycolysis WT IsC vs WT MHC **p = 0.0039, Basal glycolysis WT MHC vs WT + SHP inhibitor *p = 0.047, Glycolytic reserve WT MHC VS WT + SHP inhibitor *p = 0.0482, Max glycolysis WT IsC vs WT MHC ***p = 0.0008, Max glycolysis WT MHC VS WT + SHP inhibitor **p = 0.0015; **e** Glu/Oligo/2DG injection cd31^*−*/*−*^ MHC + SHP Inhibitor vs all ****p < 0.0001; **f** basal glycolysis *cd31*^*−/−*^ MHC vs *cd31*^*−/−*^ MHC + SHP inhibitor **p = 0.019, glycolysis reserve cd31^*−*/*−*^ MHC vs cd31^*−*/*−*^ MHC + SHP inhibitor ***p = 0.0003, max glycolysis *cd31*^*−/−*^ MHC vs *cd31*^*−/−*^ MHC + SHP inhibitor *0.0384; **i** FCCP/Rotenone injection *cd31*^*−/−*^ MHC + SHP inhibitor vs all ****p < 0.0001, FCCP/Rotenone injection *cd31*^*−/−*^ Isc vs *cd31*^*−/−*^ IsC+SHP inhibitor ***p = 0.0006; **j** max respiration *cd31*^*−/−*^ IsC vs *cd31*^*−/−*^ MHC *p = 0.0177, Max respiration *cd31*^*−/−*^ IsC vs *cd31*^*−/−*^ MHC + SHP inhibitor ***p = 0.0004, spare capacity *cd31*^*−/−*^ IsC vs *cd31*^*−/−*^ MHC ****p < 0.0001, *cd31*^*−/−*^ IsC vs *cd31*^*−/−*^ MHC + SHP inhibitor ****p < 0.0001.

The data obtained with CD31^Y686F^ (Fig. 2f, g) implicate SHP-2 activation in CD31-mediated barrier recovery along with previous observations^37^. We, therefore, examined the effect of pharmacologic inhibition of SHP-2 on the metabolic response of WT EC to MHC-stimuli. In steady-state conditions, EC monolayer permeability was increased by pre-treatment with a selective SHP1/2 small-molecule-inhibitor, without induction of cell death (Fig. 5a, b). The glycolytic response to MHC-triggering was abrogated in confluent monolayers of CD31-expressing EC following SHP inhibition (Fig. 5c, d). An even greater effect was observed when CD31-deficient EC were analyzed (Fig. 5e, f). In these experiments, the glycolytic pathway was impaired also in unstimulated EC, suggesting that additional SHP phosphatases might contribute to sustain glycolysis in quiescent endothelium. Mitochondrial respiration was not affected by SHP-inhibition in quiescent WT or CD31-deficient EC, suggesting that the defect in OXPHOS observed in *cd31*^*−/−*^ EC is not caused by lack of SHP activation. SHP inhibition did not modify OCR in MHC-stimulated WT EC (Fig. 5g, h), while it was further increased in *cd31*^*−/−*^ endothelium (Fig. 5i, j). Thus, SHP activation is required for glycolysis while it does not affect mitochondrial respiration.

### CD31 signals inhibit FoxO1 nuclear-translocation

Cellular ability to activate glycolysis depends on the availability of glycolytic enzymes^31,33^. We, therefore, investigated expression of enzymes of the glycolytic pathway known to be functional in the endothelium before and after MHC-stimulation of WT and CD31-deficient EC.

**Fig. 6:**
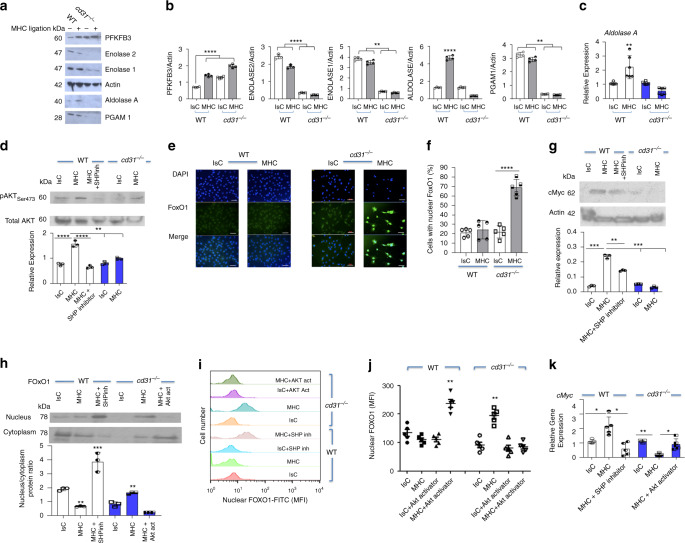
CD31 signals induce transcription of glycolytic enzymes via inhibition of FoxO1 activity. WT and *cd31*^*−/*^^−^ EC were stimulated by MHC antibody-ligation or treated with an isotype-matched control and secondary antibody (4 h). **a**, **b** Expression of the indicated enzymes was analyzed by immunoblotting and quantified by densitometry. *N* = 3 independent experiments. **c** Expression of aldolase A mRNA in WT and CD31 deficient EC was determined by RT-PCR 2 h after stimulation. *N* = 3 independent experiments. Data are mean ± SD. One-way Anova with Tuckey post-hoc test. **b** PFKFB3 WT IsC vs WT MHC ****p < 0.0001, PFKFB3 WT IsC vs *cd31*^*−/−*^ MHC ****p < 0.0001, PFKFB3 WT MHC vs *cd31*^*−/−*^ MHC ****p < 0.0001, Enolase2 WT IsC vs WT MHC ****p < 0.0001, Enolase2 *cd31*^*−/−*^ IsC vs *cd31*^*−/−*^ MHC ****p < 0.0001, Enolase1 WT IsC vs WT MHC **p < 0.009, Enolase1 *cd31*^*−/−*^ IsC vs *cd31*^*−/−*^ MHC **p < 0.003, Aldolase WT MHC vs all **** < 0.0001, PGAM1 WT IsC vs WT MHC **p = 0.007, PGAM1 *cd31*^*−/−*^ IsC vs cd31^*−*/*−*^ MHC **p = 0.0051. **c** WT MHC vs all **p = 0.001. **d** Phosphorylation of Akt (ser473) in WT and *cd31*^*−/*^^−^ EC was measured 30 min after MHC molecule triggering. WT EC were also exposed to the SHP1/2 inhibitor during stimulation. The bar graph shows the nucleus/cytoplasm ration of protein quantified by densitometry. *N* = 3 independent experiments. Data are mean ± SD. One-way Anova with Tuckey post-hoc test. WT IsC vs WT MHC ****p < 0.0001, WT MHC vs WT MHC + SHP inhibitor ****p < 0.0001,WT MHC vs *cd31*^*−/−*^ IsC **p = 0.0024, WT MHC vs *cd31*^*−/−*^ MHC **p = 0.002. **e** 2 h after stimulation EC were stained using rabbit anti-mouse FoxO1 (green) and DAPI (blue). The nuclear distance between MHC-stimulated *cd31*^*−*^*/*^−^ ECs reflects endothelial contraction, as experiments were performed starting from identical EC monolayers. Scale bar = 20 μm. *N* = 3 independent experiments. **f** For quantification, 500 cells per coverslip were analyzed, and the bar graph shows the percentage of cells displaying nuclear FoxO1 localization measured in three independent experiments ± SD. one-way Anova with Tuckey post-hoc test. cd31^*−*/*−*^ IsC vs cd31^*−*/*−*^ MHC ****p < 0.0001. **g** cMyc protein expression in WT and *cd31*^*−/−*^ EC was measured 2 h after MHC molecule triggering. WT EC were also exposed to the SHP1/2 inhibitor during stimulation. The bar graph shows protein quantification by densitometry in three independent experiments ± SD. One-way Anova with Tuckey post-hoc test. WT IsC vs WT MHC ***p = 0.0002, WT MHC VS WT MHC + SHP inhibitor **p = 0.0034, WT MHC vs *cd31*^*−/−*^ IsC ***p = 0.00023, WT MHC vs *cd31*^*−/−*^ MHC ***p = 0.0002. **h** Following antibody stimulation for 2 h nuclear fractions were isolated from EC, and subjected to anti-FoxO1 immunoblot assay. The bar graph shows protein quantification by densitometry in three independent experiments ± SEM. One-way Anova with Tuckey post-hoc test. WT MHC vs all **p = 0.0023, WT MHC + SHP inhibitor vs all ***p = 0.0009, *cd31*^*−/−*^ vs all **p = 0.007. WT EC were also exposed to the SHP1/2 inhibitor and CD31-deficient EC were pre-treated with an Akt activator (500 nM) for 3 h before stimulation (*N* = 2). In similar experiments, isolated nuclei were stained with anti-Foxo1 antibody and analyzed by flow cytometry. Representative histograms and a summary of five independent experiments are shown in (**i**, **j**), respectively. **k** *cMyc* mRNA levels in the EC stimulated with anti-MHC or control antibodies for 2 h and treated with the indicated compounds were measured by RT-PCR. *N* = 3 independent experiments. Data are mean ± SD. one-way Anova with Tuckey post-hoc test. **j** WT IsC + AKT activator vs all **p = 0.002, cd31^*−*/*−*^ vs all **p = 0.006. **k** WT IsC vs WT MHC *p = 0.044, WT MHC vs WT MHC + SHP inhibitor *p = 0.047, *cd31*^*−/−*^ IsC vs cd31^*−*/*−*^ MHC **p = 0.0065, *cd31*^*−/−*^ MHC vs *cd31*^*−/−*^ MHC + Akt activator *p = 0.043.

MHC-stimulation of WT EC did not modify expression of enolase 1 and 2 and phosphoglycerate mutase, but led to upregulation of 6-phosphofructo-2-kinase/fructose-2,6-bisphosphatase 3 (PFKFB3, Fig. 6a, b), a key regulator of glycolysis during EC migration^31^, and Aldolase A, known to bind to actin and regulate cytoskeletal reorganization^38^. Enzymes levels were substantially diminished in *cd31*^*−/−*^ endothelium, irrespective of MHC-stimulation, with the exception of elevated expression of PFKFB3, also in unstimulated CD31-deficient EC. PFKFB3 synthesize fructose-2,6-bisphosphate (F2,6P_2_), an activator of 6-phosphofructo-1-kinase (PFK-1), which converts fructose-6-phosphate (F6P) to fructose-1,6-biphosphate (F1,6P_2_), which might reflect a compensatory mechanism.

Reduced Aldolase expression by CD31-deficient EC reflected lack of increased transcription upon MHC-triggering, suggesting that CD31-signals promote a transcriptional program (Fig. 6c). Further, in WT, but not *cd31*^*−/−*^ EC, aldolase localized in areas of actin remodeling, including the lateral borders, suggesting a role for this enzyme in the maintenance of actin anchorage to intercellular junctions (Supplementary Fig. 4a).

The forkhead box O (FoxO) transcription factors control fundamental cellular processes, including metabolism. FoxO1 is a negative regulator of vascular growth^32^ Depending on its phosphorylation status, FoxO1 shuttles between nucleus and cytoplasm. When localized to the nucleus, FoxO1 modulates gene transcription by binding to response sequences located in the promoter. We have previously shown that CD31-induced AKT-mediated phosphorylation inhibits FoxOs by preventing their nuclear localization^39^.

SHP2-mediated CD31-signals maintain Akt activation^26^, hence we confirmed that Akt phosphorylation was induced in CD31-expressing, but not-deficient EC (Fig. 6d).

We subsequently analyzed FoxO1 localization following MHC-stimulation in WT and *cd31*^*−/−*^ EC. Immunofluorescence studies revealed enriched FoxO1-signal in endothelial nuclei of MHC-stimulated *cd31*^*−/−*^ EC, but not in WT EC (Fig. 6e, f).

In EC, FoxO1 downregulates glycolytic enzyme expression by suppressing cMyc-induced transcription^32^. We show that cMyc expression was enhanced by MHC-stimulation in WT, but not *cd31*^*−/−*^ EC (Fig. 6g). Notably, cMyc controls *aldolase* gene transcription^40,41^.

Treatment of WT EC with the SHP-inhibitor led to nuclear translocation of FoxO1 (Fig. 6h, i and Supplementary Fig. 5a), Conversely, Akt activation reduced FoxO1 nuclear translocation. SHP-inhibition reduced cMyc transcription in MHC-stimulated WT EC, while AKT activation restored Myc transcription in MHC-activated, CD31-deficient EC (Fig. 6j and Supplementary Fig. 5b, c).

### β-catenin contributes to metabolic responses to MHC-signals

**Fig. 7:**
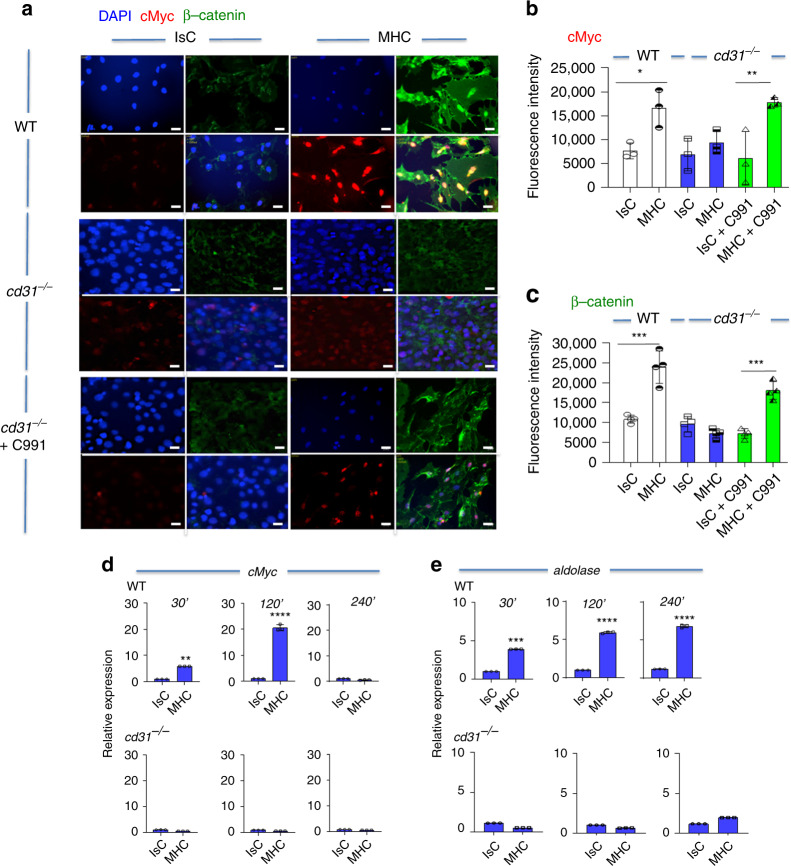
CD31 signaling promotes β-catenin nuclear translocation and upregulates cMyc expression. WT and *cd31*^*−/−*^ EC monolayers were stimulated by MHC antibody-ligation or treated with an Isotype-matched control and secondary antibodies (2 h). Some *cd31*^*−/−*^ ECs were also treated with an Akt activator (500 nM, 3 h) prior to antibody stimulation. Vehicle was added in the untreated cultures (IsC and MHC ligation). **a** β-catenin and cMyc expression were determined by immunofluorescent antibody staining and wide-field microscopy. The nucleus was stained with DAPI. The mean fluorescence intensity of cMyc and β-catenin measured in 500 cells in three independent experiments is shown in (**b**, **c**), respectively. Scale bar = 40 μm. Data are mean ± SD. one-way Anova with Tuckey post-hoc test. **b** WT IsC vs WT MHC; **c** WT IsC vs WT MHC ***p = 0.0008, cd31^*−*/*−*^ IsC + C991 vs cd31^*−*/*−*^ MHC + C991 ***p = 0.0002. **d**, **e**: *cMyc* (**d**) and *aldolase* (**e**) gene transcription by WT (upper panels) and *cd31*^*−/−*^ (lower panels) EC at the indicated time points. *n* = 3 biologically independent samples, *N* = 2 independent experiments. Error bars represent SD. One-way Anova with Tuckey post-hoc test or *T*-test (**d**, **e**). **d** WT IsC 30′ vs WT MHC 30′ **p = 0.003, WT IsC 120′ vs WT MHC 120′ ****p < 0.0001; **e** WT IsC 30′ vs WT MHC 30′ ***p = 0.0008, WT IsC 120′ vs WT MHC 120′ ****p < 0.0001, WT IsC 240′ vs WT MHC 240′ ****p < 0.0001.

In addition to stabilizing adherens junctions, β-catenin, a member of the Wnt signaling cascade, delivers signals to the nucleus, influencing metabolic transcriptional programs^42^. De-phosphorylated β-catenin can translocate into the nucleus where it promotes transcription of target genes, including cMyc^42^. Phosphorylation of β-catenin leads to ubiquitination and degradation via the proteasome^43^. CD31-signals stabilize β-catenin by preventing its phosphorylation induced by MHC-engagement (see Fig. 2b, c). To investigate whether β-catenin participates to metabolic reprogramming of EC following MHC-stimulation, localization of β-catenin in MHC-stimulated EC was investigated. MHC-ligation of WT EC enhanced β-catenin nuclear translocation leading to a concomitant increase of cMyc expression in the nucleus (Fig. 7a–c). This event did not occur in CD31-deficient EC, indicating a causative role for CD31-generated signals in the nuclear shuttling of β-catenin upon MHC-triggering. In CD31-deficient EC, nuclear localization of β-catenin and increased cMyc expression was restored following pharmacological Akt activation.

We then analyzed the transcription of both *cMyc* and its target *aldolase* upon MHC-stimulation in both WT and CD31-deficient ECs. cMyc transcription was increased as early as 30 min after stimulation, peaked at 2 h and returned to baseline by 4 h (Fig. 7d). Accordingly, aldolase transcription was also increased by 30 min, but continued to remain elevated up to 4 h after stimulation (Fig. 7e). Stimulation of *cd31*^*−/−*^ EC did not induce transcription of any of the genes analyzed.

### Inducing CD31-independent glycolysis restores EC barrier

We next investigated whether this pathway is essential to the recovery of EC barrier function following MHC-stimulation by directly activating this metabolic response independently of CD31-signals.

**Fig. 8:**
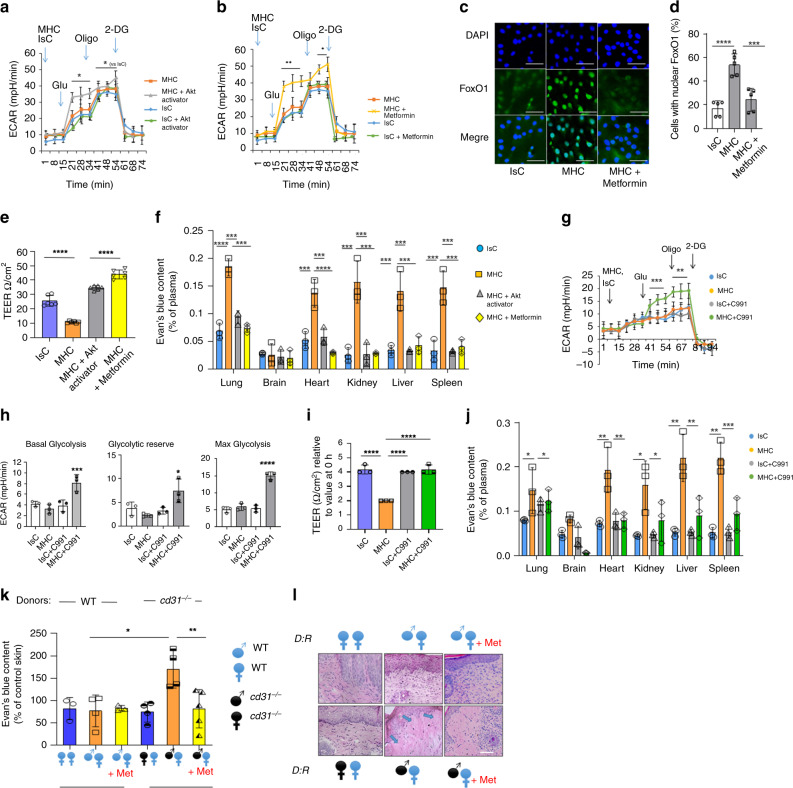
Akt and AMPK activation restore the glycolytic response in CD31-deficient EC in-vitro and in-vivo. *cd31*^*−/−*^ EC were treated with an Akt activator (500 nM, 3 h, **a**) or Metformin (5 mM, 3 h, **b**), prior to MHC-stimulation and glycolytic flux measurement *N* = 3 independent experiments. Error bars represent SD. One-way Anova with Tuckey post-hoc test. **a** Glu/Oligo injection MHC + Akt activator vs all *p = 0.04, Oligo/2DG injection MHC + Akt activator vs IsC *p = 0.048; **b** Glu/Oligo injection MHC + Metformin vs all **p = 0.003, Oligo/2DG injection MHC + Metformin vs all *p = 0.05. **c**, **d** *cd31*^*−/−*^ EC were immunostained 2 h after MHC-stimulation by rabbit anti-mouse FoxO1 (green) and DAPI (blue). In some cultures, metformin was added. 500/coverslip were analyzed. **d** Percentage of cells displaying nuclear FoxO1 localization (*N* = 3 independent experiments). Scale bar = 20 μm. **e** Quantitative analysis of TEER of *cd31*^*−/−*^ EC treated as indicated 3 h after stimulation. **f** *CD31*^*−/−*^ mice (*n* = 5 mice, *N* = 2 independent experiments) received anti-MHC and secondary cross-linking antibody (0.67 μg and 0.33 μg/kg body weight, respectively) or an Isotype-matched control antibody (IsC) and secondary cross-linking antibody (3.35 μg and 1.7 μg/kg body weight, respectively) i.v. Some mice received either an Akt activator (i.p., 7 mg/kg) or Metformin (i.p., 125 mg/kg). After 6 h, Evans blue dye (2 mg/kg) was administered by i.v. and organ dye (*n* = 3 mice; *N* = 2 independent experiments). Error bars represent SD. One-way Anova with Tuckey post-hoc test. **d** IsC vs MHC ****p < 0.0001, MHC vs MHC + Metformin ***p = 0.0004; **e** IsC vs MHC ****p < 0.0001, MHC vs MHC + Metformin ****p < 0.0001; **f** lung IsC vs MHC ****p < 0.0001, lung MHC vs MHC + Akt activator ***p = 0.0002, lung MHC vs MHC + Metformin ***p = 0.00021, heart IsC vs MHC ***p = 0.0008, heart MHC vs MHC + Akt activator ****p < 0.0001, heart MHC vs MHC + Metformin ***p = 0.00019, kidney IsC vs MHC ***p = 0.000193, kidney MHC vs MHC + Akt activator ***p = 0.000183, kidney MHC vs MHC + Metformin ***p = 0.000173, liver IsC vs MHC ***p = 0.0009, liver MHC vs MHC + Akt activator ***p = 0.00091, liver MHC vs MHC + Metformin ***p = 0.00049, spleen IsC vs MHC ***p = 0.00017, spleen MHC vs MHC + Akt activator ***p = 0.00016, spleen MHC vs MHC + Metformin ***p = 0.00027. **g**, **h** 991 AMPK-activator-treated *cd31*^*−/−*^ EC were 500 nM, 3 h), underwent MHC-stimulation and glycolytic flux measurement (**g**). **h** Basal glycolysis, glycolytic reserve and max glycolysis. **i** TEER of CD31-deficient EC treated as indicated 3 h after MHC-stimulation with or without the AMPK-selective activator 991 (500 nM). *N* = 3 independent experiments. **j** CD31^−/−^ mice (*n* = 5, *N* = 2) received anti-MHC and cross-linking antibody (0.67 μg and 0.33 μg/kg, respectively) or an IsC and cross-linking antibody (3.35 μg and 1.7 μg/kg, respectively) i.v. Some mice received AMPK-activator C991 (i.p. 7 mg/kg). After 6 h, vascular leakage was measured as above. (*n* = 3 mice, *N* = 2 independent experiments) Error bars represent SEM. One-way Anova with Tuckey post-hoc test. **g** Glu/Oligo injection MHC + C991 vs all ***p = 0.0002, Oligo/2DG MHC + C991 vs all **p = 0.0024; **h** basal glycolysis MHC + C991 vs all ***p = 0.0002, max glycolysis MHC + C991 vs all ****p < 0.0001; **i** IsC vs MHC ****p < 0.0001, MHC vs IsC + C991 ****p < 0.0001, MHC vs MHC + C991 ****p < 0.0001; **j** lung IsC vs MHC *p = 0.0309, lung MHC vs MHC + C991 *p = 0.041, heart IsC vs MHC **p = 0.0041, heart MHC vs MHC + C991 **p = 0.0058, kidney IsC vs MHC *p = 0.0205, kidney MHC vs MHC + C991 *p = 0.0338, liver IsC vs MHC **p = 0.0019, liver MHC vs MHC + C991 *p = 0.0088, spleen IsC vs MHC **p = 0.0029, spleen MHC vs MHC + C991 ***p = 0.0009. **k**, **l** WT female mice received WT (blue symbols) or *cd31*^*−/−*^ (black symbols), male or female-derived skin grafts. Some recipients were treated with Metformin (+ Met, i.p. 125 mg/kg daily), or vehicle alone. Two weeks later skin graft vascular leakage was measured as above, normalized to non-grafted skin. **l** Representative HE-stained sections of grafts harvested 2 weeks after transplantation. Arrows indicate eosinophilic (protein-rich) edema. (*n* = 3 mice, *N* = 2 independent experiments). Error bars represent SEM. Error bars represent SD. One-way Anova with Tuckey post-hoc test.  WT WT vs cd31^*−*/*−*^ WT *p = 0.032, cd31^*−*/*−*^ WT vs cd31^*−*/*−*^ WT + Met **p = 0.0098.

First, as Akt activation is instrumental to couple CD31 signals with metabolic reprogramming of EC towards glycolysis, we assessed the effect of pharmacological Akt activation on the glycolytic flux in MHC-activated CD31-deficient endothelial cells. As shown in Fig. 8a and Supplementary Fig. 6a–c, direct Akt activation could bypass the lack of CD31 signals and enhance *cd31*^*−/−*^ EC glycolysis in response to MHC-ligation.

Although via different pathways, cMyc and AMP kinase (AMPK) influence similar cellular metabolic pathways including glycolysis and oxphos^44–47^. Besides promoting glycolysis^48^, AMPK has been implicated in angiogenesis following ischemia^49^ and can induce phosphorylation and degradation of FoxO1 in EC^50^. CD31-deficient EC exposed to the AMPK-activator Metformin^50^ recovered their glycolytic response to MHC-ligation (Fig. 8b and Supplementary Fig. 6a–c). Further, treatment with Metformin restored FoxO1 nuclear exclusion in MHC-stimulated, CD31-deficient EC (Fig. 8c, d). Neither Akt- nor AMPK-activation modified mitochondrial respiration (Supplementary Fig. 6d, e).

We then assessed the impact of Akt- or AMPK-activation on the barrier response in CD31-deficient endothelium. Both Akt- and AMPK-activation restored TEER in *cd31*^*−/−*^ EC following MHC-stimulation **(**Fig. 8e**)** without affecting permeability of unstimulated WR or *cd31*^*−/−*^ EC (Supplementary Fig. 6f). Consistently, pathological vascular leakage induced by systemic MHC-ligation in *cd31*^*−/−*^ mice was corrected by the administration of either an Akt activator or Metformin (Fig. 8f).

To confirm the ability of AMPK-induced glycolysis in recovering EBF independently of CD31-signals and exclude potential off-target effects of Metformin and CD31-deficiency in leukocytes^13^, we generated bone marrow (BM) chimeras in which WT BM was administered to sublethally irradiated *cd31*^*−/−*^ recipients (CD31 selectively-expressed by EC). Some mice were treated with the high affinity AMPK-selective small molecule allosteric activator 991^51^. Treatment with AMPK activator restored *cd31*^*−/−*^ EC glycolytic response to MHC-stimulation (Fig. 8g, h), TEER recovery in MHC-stimulated *cd31*^*−/−*^ EC in-vitro (Fig. 8i) as well as barrier response following systemic MHC-stimulation in *cd31*^*−/−*^ mice (Fig. 8j).

Finally, we investigated the physiopathological relevance of our findings both in mice and humans.

To confirm that a pathway initiated by CD31-signals and inducing glycolysis is physiologically relevant to the recovery of microvascular integrity following MHC-triggering by migrating T lymphocytes, we assessed the EBF and histopathological features of rejection of male-derived CD31^−/−^ skin grafts by WT female recipients. Re-establishment of endothelial continuity between recipient and graft vasculature in HY-mismatched skin graft combinations is established by 14 days after grafting, while T-cell dependent rejection becomes clinically evident at day 28-35 and it is accelerated in CD31-deficient skin^39,52^.

As shown in Fig. 8k, l, CD31-deficient, male-derived skin was grafted onto female WT littermates and, as a control, WT females received CD31-deficent or WT, female-derived skin. Some mice were treated with Metformin (+Met), or vehicle alone. Two weeks later, mice were injected with Evans Blue i.v. and tracer extravasation in the grafts was measured. As shown in Fig. 8k, CD31-deficient male-derived skin grafts displayed significantly higher vascular leakage compared to WT-derived grafts. Importantly, female-derived skin graft vasculature appeared functionally intact irrespective of CD31 expression, confirming that the leakage was caused by MHC:HY complexes ligation by allospecific T-cells. Metformin-treatment significantly reduced tracer extravasation in CD31-deficient male-derived skin grafts. Comparison of histological features revealed substantial, protein-rich papillary dermal edema—as demonstrated by the large quantity of eosinophil material deposed between cells, indicative of severe microvascular leakage—in CD31-deficient compared to WT male-derived skin grafted onto female WT recipients (Fig. 8l). Edema in CD31-deficient grafts was reduced by treatment with Metformin. WT and CD31-deficient, female-derived skin graft did not display pathological features.

In humans, we correlated the effect of loss of CD31 expression and vascular leakage by comparing soluble CD31 (sCD31) molecules in the serum of patients with sepsis and septic shock (Supplementary Tables 1 and 2, respectively)^53^. CD31-deficient mice have severely reduced survival to LPS-induced endotoxic shock, associated with enhanced vascular permeability^16,17^. In humans, CD31 is shed from endothelium activated by strong pro-inflammatory stimuli^54,55^, and sCD31 levels have been correlated with sepsis severity^56^. As shown in Supplementary Fig. 6i, serum levels of sCD31 were significantly elevated in patients with septic shock compared with those of sepsis patients, consistent with the hypothesis that loss of CD31 expression by the endothelium undermines its ability to preserve vascular integrity.

## Discussion

**Fig. 9:**
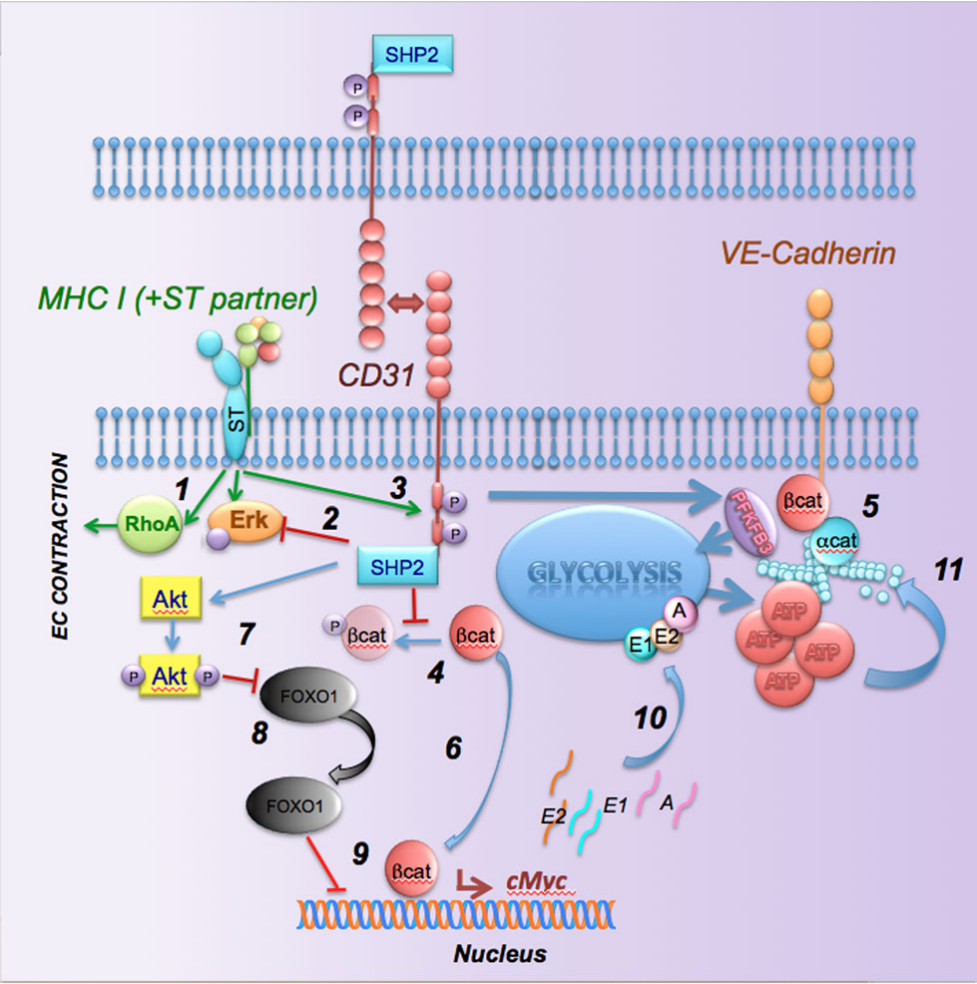
A model for the CD31-induced barrier response. MHC triggering induces RhoA and Erk activation and EC contraction (1). Erk phosphorylation is modulated by CD31 signals, possibly via SHP-2 (2). MHC signals induce CD31 ITIM phosphorylation and SHP-2 recruitment. SHP-2 prevents the phosphorylation of b-catenin (5) and VE-cadherin (6), thus stabilizing the junctional complex. In addition, dephosphorylated b-catenin can transfer to the nucleus where it induces cMyc transcription. In parallel, SHP-2 induces AKT activation which in turn inhibits FoxO1 nuclear translocation, thus preventing inhibition of cMyc transcription. This leads to enhanced transcription of glycolysis enzymes and enhanced glycolysis required for actin remodeling and maintenance of junctional anchorage.

By modeling endothelial cell contraction and vascular leakage induced by MHC-signals, we show that recovery of endothelial integrity involves a complex integration of Akt-dependent molecular events, including nuclear exclusion of FoxO1, β-catenin stabilization and nuclear translocation, and induction of cMyc expression, leading to engagement of the glycolytic flux (summarized in Fig. 9). These events likely reflect the requirement for ATP-sustained actin remodeling to re-establish junctional anchorage to cytoskeletal components. The dramatic metabolic impairment due to loss of CD31 expression selectively applies endothelial adaptation in response to stimuli that induce EC contraction and barrier breach. Previous studies have demonstrated that endothelial migration and proliferation—which also involve intense cytoskeleton reorganization—are dependent on a similar metabolic response^31,32^. Other receptors on CD31-deficient EC can induce metabolic reprogramming—as vascular functions such as angiogenesis and endothelial proliferation, which are highly dependent on glycolysis, appear to be normal in these mice.

Our observations suggest that MHC-signals p*er se* can induce stress fiber formation independently of CD31-induced signals, possibly via a pathway involving RhoA and Erk activation^22^, but that the maintenance of association of the cytoskeleton with junctions and junctional integrity require CD31 phosphorylation.

We further show that SHP-2 activation by CD31 signals is instrumental to the recovery of junctional stability, through the initiation of Akt-dependent metabolic reprogramming required for optimal cytoskeletal reorganization and junction re-assembly. The role of CD31 in the maintenance of junctional stability has been attributed to mechanical bridging of EC and junction stabilization by de-phosphorylation of β-catenin and VE-cadherin^57^. Our observations rather support a prominent role for this receptor in the initiation of a signaling pathway culminating in the production of energy to sustain actin remodeling and junction annealing.

Akt activation is central to this function of the CD31 receptor. The two major mediators, which define the contribution of Akt signaling in metabolic reprogramming leading to junction stabilization, are β-catenin and FoxO1. FoxO1 is prevented from translocating to the nucleus after phosphorylation by Akt^58^. Concomitantly, Akt inactivates GSK-3β, which sequester β-catenin in the cytoplasm^59^ leading to β-catenin stabilization and nuclear translocation^60^. Ultimately, CD31 maintains the efficiency of the glycolytic machinery in EC by promoting *β*-catenin-mediated c-Myctranscription, which in turn promotes expression of the glucose transporter Glut-1 and Aldolase A^40^.

Interestingly, in the absence of CD31 signals, PFKFB3 protein expression by EC is increased, suggesting that the regulation of this enzyme transcription is cMyc independent. PFKFB3 gene expression has been shown to be inhibited by shear stress through Kruppel-like factor-2 (KLF2) in EC^61^, and was enhanced by HIF Serum Response Factor in various tumor cell lines^61^, but has never been associated with cMyc-induced transcription.

Unlike what was observed in human EC transduced with a gain-of-function FoxO1 allele rendering FoxO1 constitutively nuclear^32^, our data indicate that enhanced FoxO1 nuclear translocation leads to increased oxidative metabolism, probably fueled by fatty acid oxidation (FAO). The reason for this discrepancy is at present unclear, but could be related to phosphatase targets other than the FoxO1 pathway in our system. It is worth noting that the identity of phosphatase enzyme(s), which dephosphorylate FoxO1 is at present unknown. In addition, the pattern of FoxO phosphorylation can differentially affect its activities^62^ and it is possible that different phosphorylation patterns might reflect a stimulus-specific modulation which leads to stimulus specific-effects.

An unexpected feature of CD31-deficient EC is their severely compromised mitochondrial respiration associated with a large number of enlarged mitochondria. It has been suggested that in response to stressors, mitochondria become enlarged decreasing the rate of oxygen consumption, leading to decreases in the rate of ROS production and ATP^63^. Relevant to the focus of this study, metformin-induced AMPK activation restores the EBF and glycolysis without affecting mitochondrial respiration, suggesting that oxidative phosphorylation is not required for restoring endothelial continuity after contraction and barrier breach. In addition, SHP inhibition does not affect OCR by WT ECs, thus ruling out a role for this CD31-signaling mediator. Understanding the molecular mechanism and the functional consequences of the defective oxphos in CD31-deficient EC will require further investigations. Of note, oxidative phosphorylation is plays a role in sustaining endothelial cell division^64^ and attenuation of vascular development has been observed in CD31-deficient mice^65^.

We further show that, unlike MHC-stimulation, exposure to insulin results in an increased ATP levels in CD31-deficient EC, suggesting that CD31-deficient EC are capable to engage the glycolytic pathway in response to insulin receptor, but not MHC-stimulation. This also implies that the metabolic alterations of CD31-deficient EC might become apparent only as a result of selected signals, such as MHC-triggering or histamine, and that insulin receptor signals are not affected by CD31 activity. The enhanced ATP production in response to insulin by CD31-deficient EC compared to WT also indicates that compensatory mechanisms are in place to compensate for the severe deficiency in mitochondrial respiration in these cells.

The contribution of CD31 signals in the maintenance of barrier integrity has long been contentious^57^. In inflammatory conditions, the impact of CD31 activity on junction stability during leukocyte migration appears to be stimulus-specific^66^. Similarly, endothelial contraction is not required for IL-1β-induced leukocyte migration^67^. Genetic deletion of the actin nucleation-promoting factor cortactin in mice is associated with reduced neutrophil recruitment but increased vascular permeability in-vivo^68^, thus demonstrating that transendothelial migration and vascular leakage can be associated, but not necessarily coupled. In agreement with these reports, our data show that ICAM-1-mediated signals do not result in endothelial contraction and temporary vascular leakage, while suggesting that metabolic adaptation by EC is paramount to the barrier response to severe ‘breaching’ signals, such as those induced by histamine, thrombin and MHC-ligation. Physiologically, this would reflect a prominent role of CD31 signaling during inflammatory conditions characterized by high endothelial contractility (histamine, MHC-triggering) leading to potential vascular damage.

CD31-induced metabolic responses appear to be largely dependent on a specific transcriptional program. While our fluxometry studies show that an increased glycolytic rate immediately follows MHC-stimulation of WT EC and MHC-stimulation rapidly induces translocation to Glut1 to the endothelial surface, full re-establishment of endothelial integrity in-vivo (see Fig. 1h, i) requires ~2–3 h. Such a prolonged energy requirement is likely to require, as we show, a rapid and sustained transcriptional reprogramming leading to increased expression of glycolysis-promoting genes such as cMyc and aldolase.

In further support of the requirement of endothelial metabolic adaptation in inflammatory conditions characterized by severe barrier breach, treatment with Metformin has been known to promote favorable outcome in sepsis, a condition characterized by systemic endothelial leakage^69–71^. Similarly, inhibition of Abl family kinases, which phosphorylate β-catenin, attenuates vascular leakage induced by thrombin, histamine, vascular endothelial growth factor (VEGF), lipopolysaccharide (LPS), and oxidative stress^72–76^. The present study provides a molecular basis for these clinical observations. Thus, in-depth knowledge of endothelial metabolic adaptation in health and disease, and of the endothelial receptors regulating metabolic reprogramming might provide novel targets for the prevention and therapy of diseases characterized by endothelial dysfunction.

## Methods

### Mice

*cd31*^*−/−*^^77^ and wild type (WT) male and female mice were bred in house in SPF conditions and used at the age of 8–10 weeks. All in-vivo experiments were conducted with strict adherence to the Home Office guidelines (PPL P71E91C8E) following approval by the Queen Mary University of London Ethics committee. The number of animals required to obtain statistical significance was estimated based on similar studies previously performed. Animals were not randomized, and no blinding was done.

### Patients

A total of 13 patients admitted to ITU (Campus Biomedico Hospital, Rome, Italy) in November–December 2018 were divided into two groups: the sepsis group (*n* = 7) and the septic shock group (*n* = 6), diagnosed based on the criteria proposed at the American College of Chest Physicians/Society of Critical Care Medicine Consensus Conference in 1992. Informed written consent was obtained from all subjects or patients’ surrogates. This study was approved by the local Research Ethics Committee (Prot. 28.18TS ComEt CBM). Serum samples were used for the sCAM analysis. The tubes were left for 20 min to allow for clotting and centrifuged at 15,609 × *g* for 10 min, at 4 °C. Serum was stored at −80 °C until analysis using a Human CD31 ELISA Kit.

### Regents

The following antibodies were used in this study: Rabbit-anti-mouse-Phospho-Akt (Ser473) Antibody (Cell signalling, AB329825) 1:200 dilution, Rabbit-anti-mouse-Akt (pan) (C67E7) Antibody (Cell signaling AB915783) 1:200 dilution, Rabbit-anti-mouse- P-ERK1/2 Antibody (Cell signalling, AB331646) 1:100 dilution, Rabbit-anti-mouse ERK1/2 Antibody (Santa Cruz, Sc292838) 1:100 dilution, Mouse-anti-mouse P-tyrosine (PY20) Antibody (Santa Cruz, Sc 508) 1:200 dilution, Rabbit-anti-mouse SHP2 Antibody (Abcam ab131541) 1:200 dilution, Rabbit-anti-mouse Ubiquitin Antibody (Abcam ab7780) 1:200 dilution, Goat anti-actin-I19 Antibody (Santa Cruz Biotechnology, Sc1616) 1:200 dilution, Rabbit-anti-mouse PFKFB3 (D7H4Q) Antibody (Cell signalling, 13123) 1:200 dilution, Rabbit-anti-mouse Enolase-2 (D20H2) Antibody (Cell signaling, AB11178392) 1:200 dilution, Rabbit-anti-mouse Enolase-1 Antibody (Cell signalling, AB2246524) 1:200 dilution, Rabbit-anti-mouse Aldolase A Antibody (Cell signalling, AB2226674) 1:200 dilution, Rabbit-anti-mouse PGAM1 Antibody (Cell signalling,12098) 1:200 dilution, Anti-rabbit IgG, HRP-linked Antibody (Cell signalling, AB2099233) 1:200 dilution, Rabbit-anti-mouse rac1/cdc42 Antibody (Cell signalling, AB10612265) 1:200 dilution, Rabbit-anti-mouse PE-conjugated anti- CD31 Antibody (Thermofisher AB465631) 1:200 dilution, Rat IgG2a K Isotype Control Antibody (Thermofisher = AB470104), Rabbit-anti-mouse p-β-catenin (phospho Y654) Antibody (Abcam ab59430) 1:200 dilution, Rabbit-anti-mouse VE-cadherin (phospho Y685) Antibody (Abcam AB10971838) 1:200 dilution, Rabbit-anti-mouse VE-cadherin Antibody (Abcam ab205336) 1:200 dilution.

Rabbit-anti-mouse β-Catenin Antibody (Abcam AB11127855) 1:200 dilution,Rabbit-anti-mouse Anti-PFKFB3 Antibody (Abcam AB181861) 1:200 dilution, Rabbit-anti-mouse Anti-beta Actin Antibody (Abcam AB16039) 0.5 µg/mL dilution, Rabbit-anti-mouse c-Myc Antibody (Abcam AB10858578), Alexa Fluor® 555 goat anti-mouse Ig Antibody (Life Technology AB2563179), and FITC Donkey anti-rabbit IgG (minimal x-reactivity) Antibody (Life Technology AB893531), Rabbit-anti-mouse FoxO1 Antibody (Abcam AB2106495) 1:200 dilution, Mouse-anti-mouse anti-Glut1-PE Antibody (Novusbio, NB110-39113PE) 1:200 dilution, Rabbit-anti-mouse anti-Erk1/2 (H72) Antibody (Santa Cruz, SC292838) 1:200 dilution, Rabbit-anti-mouse Glut1 Antibody AF647 (Novus Bio, NB110-39113AF647) 1:100 dilution, Rabbit-anti-mouse Aldolase antibody (Abcam, ab169544) 1:200 dilution, tetramethyl rhodamine B isothiocyanate-phalloidin (Sigma, P1951) 50 units/assay dilution.

Mouse-anti-mouse H-2Ld/H-2Db (MHC-class-I) Antibody (Biolegend, 114502), Rat-anti-mouse IgG, (Biolegend, 553445), (Blocking) rat anti-mouse CD31 clone 390 (eBioscience, AB10060377), (Stimulate) rabbit anti-mouse CD31 (Abcam, ab2836), Goat–anti-rabbit Ig Antibody, (Agrisera, AS10 665), (cross-linkage) Mouse-anti-mouse ICAM-1 (Abcam, ab2213) were used to stimulate MHC molecules on the endothelium.

Other reagents include the SHP1/2 inhibitor CAS 56990-57-9, (Bioscience, NSC 87877), Akt activator (Tocris, SC79), Metformin (Sigma-Aldrich, 317240), Evans blue (Sigma-Aldrich, E2129-10G), 3-(Trimethylsilyl)propionic-2,2,3,3-d4 acid sodium salt (Sigma-Aldrich, 269913-1G), Tetramethyl rhodamine B isothiocyanate–conjugated phalloidin (Sigma-Aldrich, P1951), ProLong Gold Antifade (Life Technologies, P36930), Type IV Collagenase (Sigma-Aldrich, C5138), FBS (Seralab, A210009), Dulbecco’s Modified Eagle media (DMEM, Gibco 41966-052), glutamine (Gibco250-30), 2-mercaptoethanol (2-ME) (Gibco 31350-010), sodium pyruvate (Gibco 11360-039), HEPES (Gibco 15630-056), non-essential amino acids (Gibco 11140-050),trypsin/EDTA (Gibco T4049), murine IFN-γ (PeproTech 315-05), glucose free T-cell medium (Gibco, 11879-020), 6-NBDG (Life Technologies, N23106), RNeasy Mini Kit (50) (Qiagen, 74104), iQ™ SYBR® Green Supermix (Biorad, 1708880), High-Capacity RNA-to-cDNA™ Kit (Life Technologies, 4387406), MitoTracker™ Green FM (Thermofisher Scientific, M7514),MitoTracker™ Red CMXRos (Thermofisher Scientific,M7512), Uranyl acetate (VWR 102092-282), Histamine (Sigma, 59964), Corning® HTS Transwell®-24 well permeable supports (Fisher Scientific Ltd, 10228861), Formamide (Sigma, F9037), Paraformaldehyde 95% (Sigma, 158127), Staphylococcal enterotoxin A from Staphylococcus aureus (Sigma, S9399), 2-Deoxy-D-glucose (Sigma, D8375), Trypsin-EDTA (0.25%) phenol red (Thermo Fisher 25200056), ECL Western Blotting Detection Reagent (Amersham, RPN2209), Fujifilm Super RX film (Fuji, RF12), Foxp3/Transcription Factor Staining Buffer Set (Thermo Fisher, 00-523-00), Poly-D-Lysin (Gibco™, A3890401), Insulin (Sigma, 11061-68-0).

### Isolation and culture of primary microvascular endothelial cells

Microvascular endothelial cells were isolated from murine lungs^20^ and cultured in (DMEM ThermoFisher 41966), supplemented with 10% FCS). When confluent, cells were detached with trypsin/EDTA (Gibco, T4049) and passaged. Cells were used for up to 4 passages in culture.

### Antibody-mediated EC activation

Monolayers of WT EC were stimulated with 1 μg/ml mouse–anti-mouse H-2Ld/H-2Db (BD Biosciences 14-5999-85) or isotype control and 0.5 μg/ml goat–anti-mouse IgG (BioLegend 405301) with and without coligation of CD31 molecules (rabbit-anti-mouse CD31 5 μg/ml plus goat–anti-rabbit Ig 2.5 μg/ml). In addition, WT EC underwent MHC class 1 molecule stimulation in the presence or absence of ICAM-1 ligation (rat–anti-mouse CD54 (eBioscience 14-0549-82) 5 μg/ml plus goat anti-rat Ig 2.5 μg/ml (Biolegend 400202).

### Measurement of trans-endothelial electrical resistance (TEER)

EC were grown on Transwell polycarbonate filters (pore size, 0.4 µm; Sigma-Aldrich, CLS3396) first coated with calfskin collagen type I (Sigma-Aldrich, C3867) and bovine plasma fibronectin (Sigma-Aldrich, F1141). Transendothelial resistance across the monolayer was determined by using an Endohmeter (World Precision Instruments, Sarasota, FL) stabilized at 148 ± 12 Ω. Resistance from coated cell-free inserts was always subtracted from the resistance obtained in the presence of endothelial cells.

### Actin cytoskeleton analysis

EC (10^5^) were seeded onto each well of 24-well plates containing glass coverslips (VWR Internationa, P24G-1.0-10-F) coated with 100 μg of 2% gelatin (Sigma-Aldrich, G9382). They were incubated overnight at 37 °C with 5% CO_2_ in EC media to form a monolayer. EC monolayers were then fixed with 4% buffered paraformaldehyde (Sigma-Aldrich, 16005) for 20 min at 4 °C, washed three times with PBS and stained with 1 ng/ml tetramethyl rhodamine B isothiocyanate–conjugated phalloidin (Sigma-Aldrich, P1951) for 30 min at 37 °C. Coverslips were extensively washed, air dried, and mounted in Vectorshield (Vector Laboratories, H-5000) mounting medium for fluorescence with DAPI (Vector Laboratories, H-1800) or Hoechst (Sigma, 94403) on glass slides. The slides were analyzed with wide-field fluorescence microscopy.

### ATP assay

ATP production by EC was measured using the ATP Assay Kit (Abcam, ab83355). 10^6^ WT and CD31 deficiency EC with or without treatments were harvested and homogenized, centrifuged to remove insoluble material, and the supernatant collected for use in subsequent steps according to the manufacturer’s instructions.

### Confocal microscopy

EC (10^5^) were seeded onto each well of 24-well plates containing glass coverslips (VWR International, P24G-1.0-10-F) previously coated with 50ug/ml Polyl-D-lysin (Gibco, A3890401). They were incubated overnight at 37 °C with 5% CO_2_ in EC media to form a monolayer. EC monolayers were then fixed with 4% buffered paraformaldehyde (Sigma-Aldrich, 16005) for 20 min at 4 °C, washed three times with PBS and stained with Glut 1 AF647 antibody (Novus Biological, NB110-39113), Aldolase 488 (Novus Biological, 42620AF488), Phalloidin 586 (ThermoFisher Scientific A12380) antibodies for 4 h at room temperature. Coverslips were extensively washed, air dried, and mounted in Vectorshield (Vector Laboratories, P24G-1.0-10-F) mounting medium for fluorescence with DAPI (Vector Laboratories, H-1800) or Hoechst (Sigma, 94403) on glass slides. The slides were analyzed with a Zeiss LSM800 confocal microscope.

### Widefield deconvolution fluorescence microscopy

EC were cultured in DMEM medium and fixed with 3.7% formaldehyde. After fixing, they were stained with the antibodies indicated in the figures. Coverslips were extensively washed, air dried, and mounted in Vectorshield mounting medium for fluorescence with DAPI (Vector Laboratories, H-1800) on glass slides. Cells were visualized using a Zeiss Z1 fluorescence microscope (Carl Zeiss) equipped with an AxioCam MRm cooled monochrome digital camera and an ApoTome.2 imaging unit. Images were acquired using a Plan Apochromat 10×/0.8 NA objective. Standard epi-illuminating fluorescein and rhodamine fluorescence filter cubes were used, and 12-bit image datasets were generated using Axiovision software version 4.8.

### Quantitative real-time polymerase-chain reaction (qRT-PCR)

Cells were harvested and stored in RNA-later (Qiagen, Crawley, UK) at –80 °C until processing. RNA was purified using Trizol reagent according to the manufacturer’s instructions (Life Technologies) and assessed for quality and quantity using absorption measurements. Reverse transcription was performed according to the manufacturer’s instruction (Applied Biosystems). Gene expression analysis was done using SYBR Green Supermix (Biorad) in CFX connect light cycler (Biorad), according to the manufacturer’s instructions. Expression was calculated using the ΔΔct method^78^ and normalized to a housekeeper gene (GAPDH). Primers for qPCR were designed with the help of online tools (Primer 3Plus) using at least one exon junction-binding site per primer pair. The sequences of the qPCR Primers are as follows:

*Glut-1* (5′-CACTGTGGTGTCGCTGTTTG-3′ and 5′-ATGGAATAGGACCAGGGCCT-3′), *VE-cadherin* (5′-TCTTGCCAGCAAACTCTCCT-3′ and 5′-TTGGAATCAAATGCACATCG-3′), c-myc (5′-GCCCAGTGAGGATATCTGGA-3′ and 5′-ATCGCAGATGAAGCTCTGGT-3′), *FoxO1* (5′-GTGAACACCATGCCTCACAC-3′ and 5′-CACAGTCCAAGCGCTCAATA-3′), *c-myc* (5′-GCCCAGTGAGGATATCTGGA-3′ and 5′-ATCGCAGATGAAGCTCTGGT-3′), mCPT-la (5′-CCAAGTATCTGGCAGTCGA-3′ and 5′-CGCCACAGGACACATAGT-3′)*, GAPDH* (5′-AGAACGGGAAGCTTGTCATCA-3′ and 5′-GACCTTGCCCACAGCCTTG-3′), *Aldolase* (5′-TGGACTAGAGGGACCTGGTG-3′ and 5′-GGGAGGGGGTAATATGGCTA -3′).

The thermal cycling profile for amplification was 95 °C for 10 min, followed by 40 cycles of 95 °C for 15 s and 54 °C for 1 min. Amplification was 95 °C for 10 min, followed by 40 cycles of 95 °C for 15 s and 60 °C for 1 min. To ensure the amplification specificity, the melting curve program was set as follows: 95 °C for 15 s, 60 °C for 1 min, and 95 °C for 15 s, right after the PCR cycles.

The qPCR data were analyzed using the delta CT method by taking the CT values of the genes of interest from the house keeping gene following by normalization to the wildtype control sample.

### Cellular Fractionation and Isolation of Nuclei

Cells (5–10 × 10^6^) were cultured in 60-mm diameter culture dishes until ~80% confluency. For isolation of nuclear extracts, the cells were then collected into microtubes, centrifuged for 20 s in a microcentrifuge, and resuspended in 200 μl of 10.0 mM Hepes (Gibco, 15630080), pH 7.9, containing 10.0 mM KCl, 1.5 mM MgCl_2_, and 0.5 mM dithiothreitol. After incubation at 4 °C for 15 min, the cells were lysed by passing 10 times through a 22-gauge needle. Next, the cells were centrifuged for 20 s in a microcentrifuge, and the supernatant, cytoplasmic fraction was collected and frozen in small aliquots. The pellet, which contained the nuclei, was resuspended in 150 μl of 20 mM Hepes, pH 7.9, containing 20% v/v glycerol, 0.1 mM KCl, 0.2 mM EDTA (Invitrogen, AM9912), 0.5 mM dithiothreitol (Thermo Scientific, A39255), and 0.5 mM phenylmethanesulfonyl fluoride (Sigma Aldrich,10837091001) and then stirred at 4 °C for 30 min. The nuclear extracts were then centrifuged for 20 min at 4 °C in a microcentrifuge. The supernatant was collected, aliquoted into small volumes, and stored at −80 °C.

### Western blotting

Nuclear or whole-cell proteins derived from each cell sample were fractionated by SDS-PAGE, blotted onto a nitrocellulose membrane (Whatman, WHA10402506). Membranes were blocked for 2 hours at room temperature in 5% milk/TBS-Tween 20 and were incubated overnight at 4 °C with the primary antibodies listed in *Reagents and Antibodies* in the main text and subsequently with HRP-conjugated secondary antibody (1:5,000; Amersham Bioscience). Films then were scanned, and the intensity of the bands was quantified using ImageJ Software v.1.37c (NIH).

### Immunoprecipitation

Cells (10^7^) were lysed in 1 mL lysis buffer [20 mM Tris⋅HCl (pH 8), 1% Triton X-100, 150 mM NaCl, 1 mM Na_3_VO_4_, 1 mM 4-benzenesulfonyl fluoride hydrochloride, 1 μg/mL leupeptin]. After centrifugation, the lysate was precleared using Protein G-Sepharose (Sigma-Aldrich, P3296) for 30 min at 4 °C and then was incubated with specific antibodies for 1 h before the addition of Protein G-Sepharose and overnight incubation at 4 °C. Samples then were washed three times with lysis buffer, boiled in SDS/PAGE sample buffer, resolved using 10% SDS/PAGE, and analyzed by Western blotting.

### In-vitro 6NBDG uptake assay

Freshly isolated EC were washed in PBS and resuspended in glucose-free cell medium (Gibco, Cat11879-020) containing various mentioned 30 6-NBDG (Life Technologies, CatN23106) in glucose free cell medium was then added to the cells and the cells were further incubated for an additional 10–15 min. Finally, the cells were washed twice with warm PBS and resuspended in flow cytometry buffer and placed on ice. Immediate analysis was performed using flow cytometry to observe fluorescence uptake by the EC.

### Measurement of ECAR and OCR

Real time bioenergetics analysis of extracellular acidification rates (ECAR) and oxygen consumption rates (OCR) of T-cells subjected to antibody stimulation was performed using the XF analyzer (Seahorse Biosciences). T-cells were cultured in serum free, unbuffered XF assay medium (Seahorse biosciences, Cat 102365-100) for 1 h. The cells were then seeded (6 × 10^5^/well) into the seahorse XF24 cell plates for analysis. Perturbation profiling of the use of metabolic pathways by T-cells was achieved by the addition of oligomycin (1 μM), FCCP (1 μM), Antimycin A (1 μM), rotenone (1 μM), D-glucose (10 mM), 2-deoxy-D-glucose (2DG, 50 mM; all from Seahorse biosciences, Cat# 103020-100 and 103015-100). Experiments with the Seahorse system were done with the following assay conditions: 2 min mixture; 2 min wait; and 4–5 min measurement. Metabolic parameters were calculated by Wave v2.4.1 Software. Experiments were done in at least triplicate wells.

### Mitochondrial oxidation of glucose, glutamine, and fatty acid oxidatio**n**

Oxidation rates of glucose, glutamine, and FAO were measured using an XFp Extracellular Flux Seahorse Analyzer (Seahorse Bioscience). *WT* and *CD31 deficiency* cells (15,000/well) were treated with MHC triggering for 45 min prior to the start of the XFp Mito Fuel Flex Test kit, which was performed in accordance with manufacturer′s instructions. Each plotted value is the mean of at least 6 replicates and is normalized to Hoechst signal in each well.

### Flow cytometry

Cells were suspended in FACS buffer (PBS, 1% BSA, 0.01% sodium azide), stained with the appropriate concentration of fluorescence-conjugated antibodies, or isotype control antibodies, according to the manufacturer’s instructions, fixed in fix buffer (PBS, 4% paraformaldehyde, 1% FCS), and analyzed by a FACSAria cell sorter (Becton Dickinson). Acquired samples were analyzed using FlowJo 7.6 software (Tree Star, Inc.).

### Measurement of endothelial permeability in-vivo

Vascular permeability was quantitatively evaluated by extravasation of Evans blue as a marker of albumin extravasation. Briefly, mice received anti-MHC and secondary cross-linking antibody (0.67 μg and 0.33 μg/kg body weight, respectively) or anti-ICAM1 and secondary cross-linking antibody (3.35 μg and 1.7 μg/kg body weight, respectively) before Evans blue dye (2 mg/kg) i.v. injection. 45 min later. As indicated, some MHC-stimulated mice received either an Akt activator (administered i.p. at a dose of 7 mg/kg) or Metformin (i.p. 125 mg/kg) before administration of the antibodies. WT and *cd31-/-* mice from treatment and control groups were deeply anesthetized with chloral hydrate, and blood was obtained by cardiac puncture. After the mice were sacrificed, organs were collected, weighted and incubated in formamide (1 ml/100 mg) for 48 hours at 56 °C to extract Evans blue. Absorbance at 600 nm was normalized by tissue weight, and by the relative concentration of Evans blue present in the blood of the corresponding mice.

### Skin grafting

Skin grafting was conducted using a method previously described by Billingham and Medawar^79^ using tail skin grafted onto the lateral thorax. Briefly, donor tail skin was removed and cut into 1 cm^2^ sections. A piece of skin was removed from the right flank of anesthetized recipient mice to create a graft bed and a 1 cm^2^ piece of tail skin was placed in the graft bed. The graft was covered with muslin and a plaster cast was then wrapped around the midriff and graft. Plasters were removed 7–10 days after grafting and grafts were inspected every other day.

### Electron microscopy of resin-embedded cells

For high-pressure freezing suspension, cultured endothelial cells were harvested by filtering and immediately frozen in a high-pressure freezing apparatus (HPF010; Bal-Tec, Balzers, Liechtenstein). For subsequent freeze substitution, the material was kept at −85 °C for 60 h before slowly being warmed to 0 °C for a period of 18 h. Substitution was performed in an AFS freeze substitution unit (Leica, Bensheim, Germany). The sections were poststained with aqueous uranyl acetate/lead citrate, and images were captured with a Hitachi H7650 transmission electron microscope (Hitachi High-Technologies) operating at 80 kV.

### Statistical analysis

Data were statistically analyzed using Prism 5.03 software (GraphPad). The statistical tests used are indicated in the corresponding Figure legends.

### Reporting summary

Further information on research design is available in the Nature Research Reporting Summary linked to this article.

## Supplementary information

Supplementary Information

Reporting Summary

## Data Availability

All data supporting the results presented herein are available from the corresponding authors upon reasonable request. The source data for all the graphs and un-cropped gels and blots in the main Figures and Supplementary Information are provided as a Source data file.
