# Supplementary material for: Preservation of microvascular barrier function requires CD31 receptor-induced metabolic reprogramming
Source: Nat Commun. 2020 Jul 17;11:3595. doi: 10.1038/s41467-020-17329-8 (PMC7367815; doi:10.1038/s41467-020-17329-8)
Supplement: Supplementary file 1 — Supplementary Information [file 41467_2020_17329_MOESM1_ESM.pdf]

SUPPLEMENTARY INFORMATION

Supplementary Figure 1

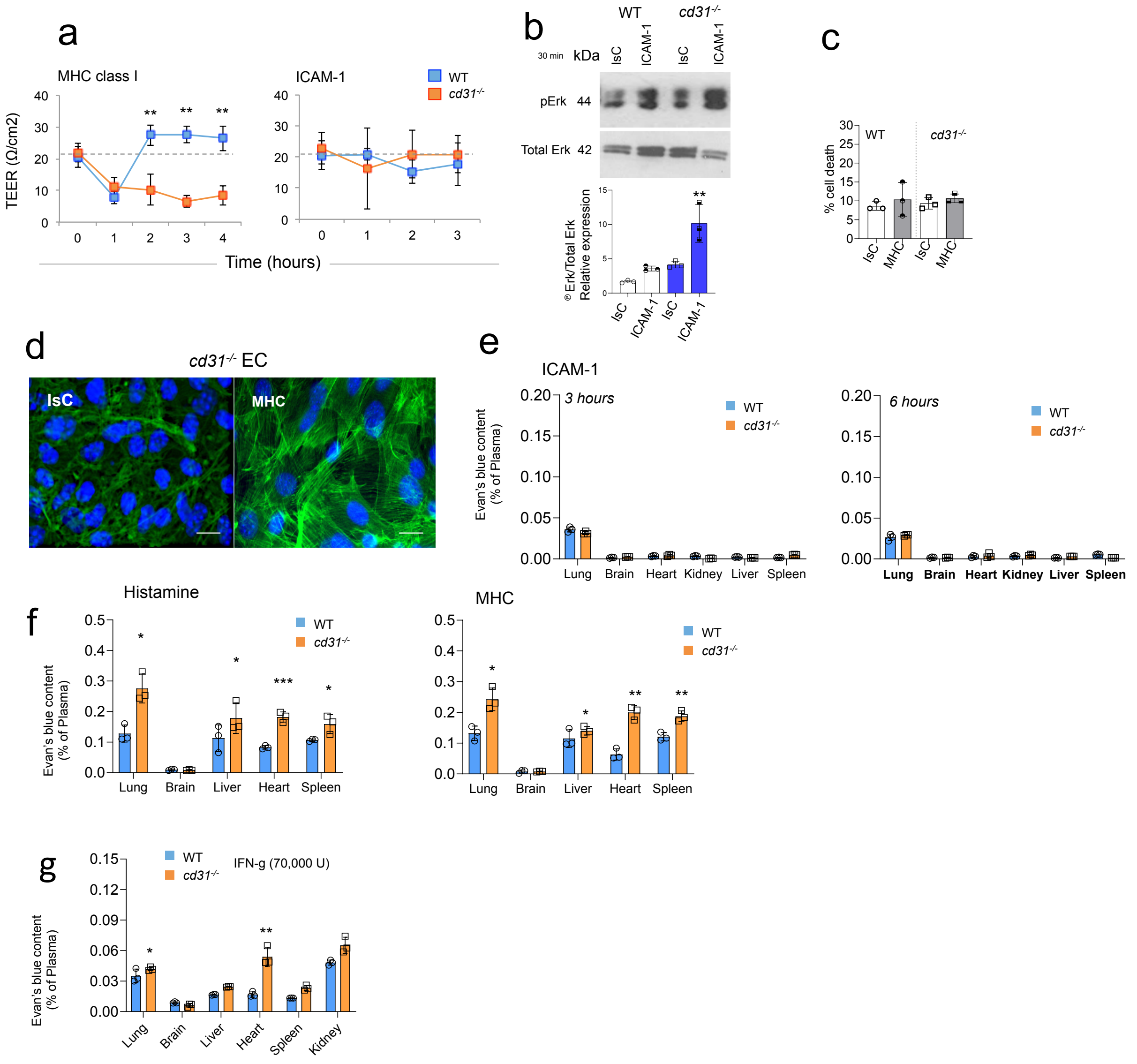

MHC, but not ICAM-1 stimulation augments endothelial permeability in vitro and in vivo. (**a**) WT and *cd31*$^{-/-}$ EC (6 × 10^4/well) previously treated with 300 U/ml IFN-γ for 48 hours (to enhance MHC molecule and ICAM-1 expression). EC were stimulated with 5 µg/ml anti-mouse H-2Ld/H-2Db, or anti-ICAM-1 or relevant isotype control followed by a secondary cross-linking

Ab. (N=2 independent experiments, n=3 biologically independent samples). The error bars represent SD. One-way Anova with Tuckey post-hoc test. 2 hours WT vs *cd31*$^{-/-}$ **p=0.0027 , 3 hours WT *vs cd31*$^{-/-}$ **p=0.00477 , 4 hours WT *vs cd31*$^{-/-}$ **p=0.00278

(**b**) Western blot analysis of Erk phosphorylation by WT or CD31-deficient EC 30 minutes after ICAM-1 stimulation. The bar graph shows relative protein expression ± SEM. N=3 independent experiments , One-way Anova with Tuckey post-hoc test. *cd31*$^{-/-}$ ICAM-1 vs WT IsC **p=0.004 , *cd31*$^{-/-}$ ICAM-1 vs WT ICAM-1 **p=0.0021 , *cd31*$^{-/-}$ ICAM-1 vs *cd31*$^{-/-}$ IsC **p=0.0037 (**c**) EC death was evaluated by 4 hours by TpB exclusion assay. The mean percentages of dead cells in three independent experiments (±SD) are shown. **p<0.01, One-way Anova with Tuckey post-hoc test.

d) Following MHC antibody-stimulation for 30 minutes, *cd31*$^{-/-}$ EC were fixed and stained with rhodamine-phalloidin. Images taken on EC monolayers seeded at identical density are shown. Scale bar, 20 μm. (N=1 experiment, n=3 biologically independent samples)

e) WT or *cd31*$^{-/-}$ mice (n=6 mice, N=2 independent experiments) received anti-ICAM1 and secondary cross-linking antibody (3.35µg and 1.7µg/kg body weight, respectively) in saline solution i.v.. After 3 h or 6 h, 100 µL of 2% Evans blue in saline solution was injected i.v.. Dye was allowed to circulate for 45 min before organs dye content was assessed spectrophotometrically and normalized to plasma levels (data are mean ± SEM). One-way Anova with Tuckey post-hoc test (WT vs cd31-/-).

f) WT or *cd31*$^{-/-}$ mice (n=6 mice, N=2 independent experiments) received histamine (60ng/mouse) or anti-MHC and secondary cross-linking antibody (0.67µg and 0.33µg/kg body weight, respectively), in saline solution i.v.. After 4 hours 100 µL of 2% Evans blue in saline solution was injected i.v.. Dye was allowed to circulate for 45 min before organs dye content was assessed spectrophotometrically and normalized to plasma levels (data are mean ± SEM). One-way Anova with Tuckey post-hoc test. Histamine lung WT vs *cd31*$^{-/-}$ *p=0.0201 , Histamine liver WT vs *cd31*$^{-/-}$ *p=0.0447 , Histamine heart WT vs *cd31*$^{-/-}$ ***p=0.0006 , Histamine spleen WT vs *cd31*$^{-/-}$ *p=0.0433, MHC lung WT vs *cd31*$^{-/-}$ *p=0.0119 , MHC liver WT vs *cd31*$^{-/-}$ *p=0.011 , MHC heart WT vs *cd31*$^{-/-}$ **p=0.0011 , MHC spleen WT vs *cd31*$^{-/-}$ *p=0.0037

Panel g lung WT vs *cd31*$^{-/-}$ *p=0.0393 , heart WT vs cd31$^{-/-}$ **p=0.0051

g) WT or *cd31*$^{-/-}$ mice (n=6 mice, N=2 independent experiments) received IFN-γ (70,000U/mouse) in saline solution i.v.. After 48 hours 100 µL of 2% Evans blue in saline solution was injected i.v.. Dye was allowed to circulate for 45 min before organs dye content was assessed spectrophotometrically and normalized to plasma levels (data are mean ± SEM). One-way Anova with Tuckey post-hoc test. Lung WT vs *cd31*$^{-/-}$ *p=0.0393 , heart WT vs *cd31*$^{-/-}$ **p=0.0051

Supplementary Figure 2

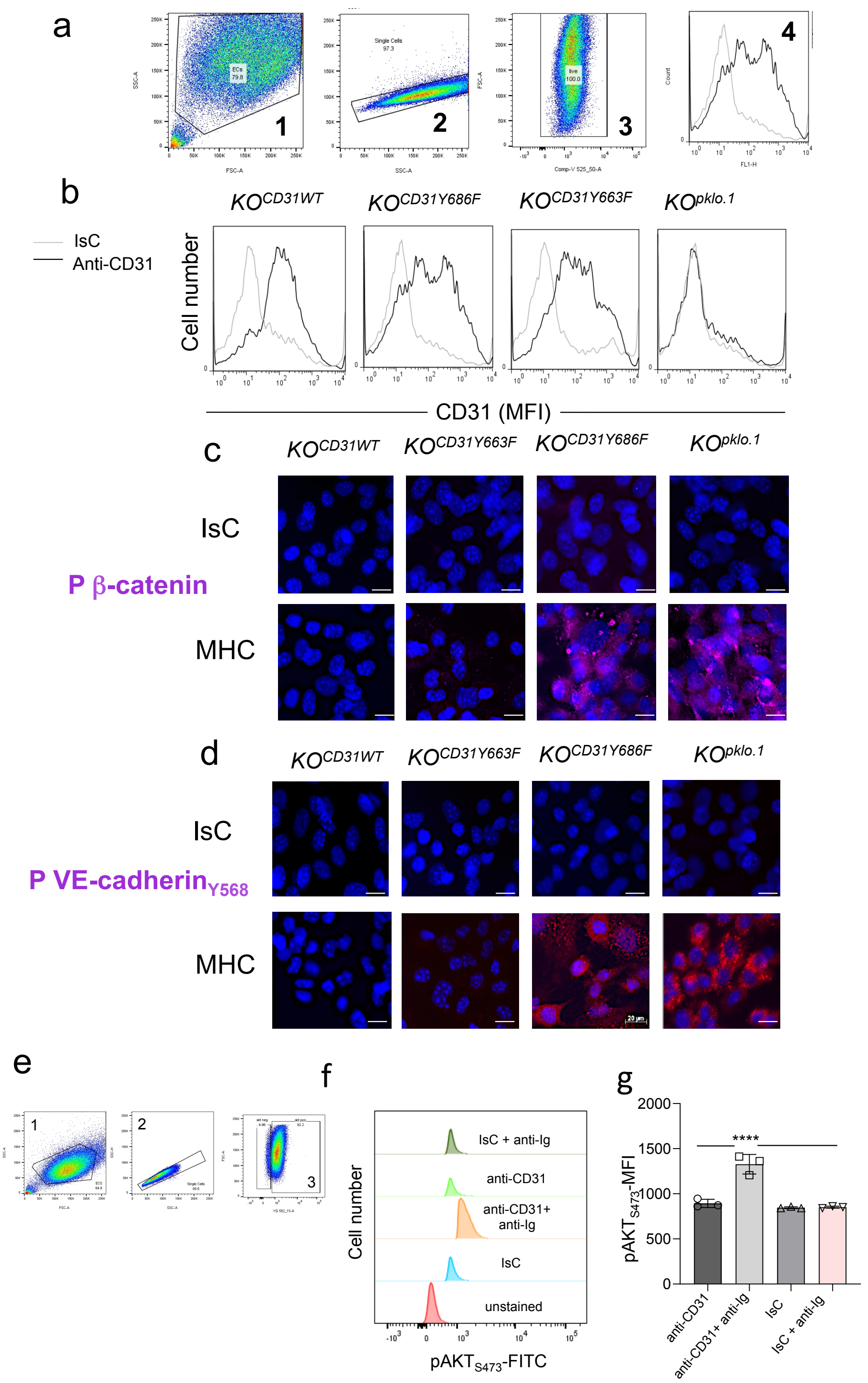

Responses by *cd31-/-* EC transduced with *CD31Y663F* or *CD31Y663F* gene constructs to MHC stimulation.

Two CD31 gene constructs were generated with mutation leading to the loss-of-function amino acid

substitutions Y663F and Y686F in the ITIMs, which were lentivirally transduced into CD31 KO ECs ($KO^{cd31Y663F}$, $KO^{cd31Y686F}$ ECs). As a control WT (KO^{CD31WT}) *cd31* constructs and plasmid ($KO^{pklo.1}$) alone were also transduced. Panel a: EC were identified by size and granularity (1) and following gating on single (2) and live (3) cells CD31 surface expression was analyzed (4). Panel b: representative histograms showing expression of the transduced genes by ECs. (N=2 independent experiments).

Panels c-d: EC (6 × 10^4/well) previously treated with 300 U/ml IFN-γ for 48 hours (to enhance MHC molecule expression). EC were stimulated with 5 μg/ml anti-mouse H-2Ld/H-2Db followed by a secondary cross-linking Ab. Expression of phosphorylated β-catenin (c) and VE-cadherin (Y568) was assessed 30 minutes later by widefield immunofluorescence microscopy. Scale bar, 20 μm. Magnification ×20. (N=3 independent experiments)

Panels e-g: EC (2 × 10^4/well) previously treated with 300 U/ml IFN-γ for 48 hours (to enhance MHC molecule expression) were stimulated with 5 μg/ml anti-mouse H-2Ld/H-2Db followed by a secondary cross-linking Ab. Expression of phosphorylated Akt (S473) was assessed 30 minutes later by flow cytometry. EC were identified by size and granularity (1) and following gating on single cells (2) pAkt expression was analyzed (3). A representative histogram stack is shown in panel f. The mean pAkt MFI measured in 3 independent experiments is shown ±SD is shown in panel g. One-way Anova with Tuckey post-hoc test. Anti-CD31vs anti-CD31+anti-Ig ****$p<0.0001$, anti-CD31+anti-Ig vs IsC+anti-Ig ****$p<0.0001$

Supplementary Figure 3

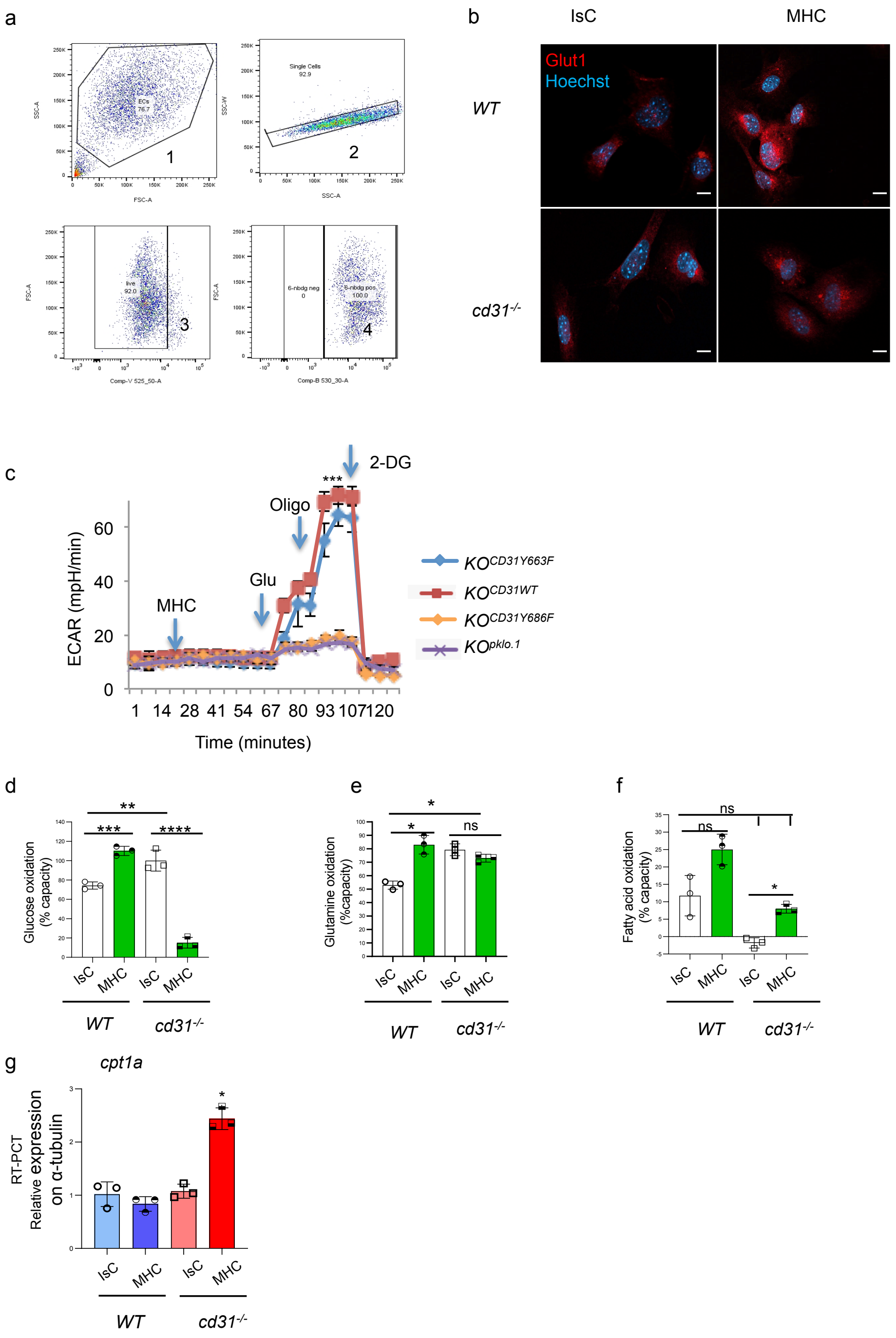

Mitochondrial fuel usage by WT and CD31-deficient EC.

(**a**) Gating strategy for analysis of 6-NBDG uptake. To analyze 6-NBDG measured by flow cytometry, EC were identified by size and granularity (1) and following gating on single (2) and live (3) cells and pAkt expression was analyzed (4).

(**b**) EC (6 × 10^4/well) previously treated with 300 U/ml IFN-γ for 48 hours (to enhance MHC molecule expression), were stimulated with 5 μg/ml anti-mouse H-2Ld/H-2Db followed by a secondary cross-linking Ab. Expression of Glut1 was assessed 30 minutes later by confocal microscopy. N=2 Scale bar, 20 μm. Magnification ×20. (N=2 independent experiments)

The extracellular acidification rate (ECAR) of antibody-stimulated KO^{CD31WT}, $KO^{cd31Y663F}$, $KO^{cd31Y686F}$ and $KO^{pklo.1}$ECs is shown in panel **c**. The error bars represent SD. (N=2 independent experiments). One-way Anova. KO^{CD31WT} vs all ***p=0.0002

Using the Seahorse XF Mito Fuel Flex Test, we measured the capacity (i.e., the ability to use a specific fuel to meet energy demand) of WT and *cd31 -/-* EC to oxidize glucose (**d**), glutamine (**e**) and fatty acids (**f**) when pre-challenged with MHC-stimulation (4 h, N=1, n=3). MHC-stimulated WT EC exhibited a significantly higher capacity to use and increase the oxidation of glutamine when trying to compensate for UK5099 and etomoxir-induced inhibition of alternative fuel pathways (i.e., glucose oxidation and long chain fatty acid oxidation, respectively). In contrast, CD31-deficient ECs exhibited a significantly lower capacity to use and increase the oxidation of glucose when trying to compensate for BPTES- and Etomoxir-induced inhibition of alternative fuel pathways (i.e., glutamine oxidation and long chain fatty acid oxidation, respectively). However, *cd31-/-* EC displayed a significant increase in fatty acid oxidation (FAO) upon MHC stimulation. Data are shown as mean ± SD. (N=3 independent experiments) One-way Anova with Tuckey post-hoc test. (**d**) WT IsC vs WT MHC ***p=0.0009 , WT IsC vs $cd31^{-/-}$ IsC **p=0.0074 , $cd31^{-/-}$ IsC vs $cd31^{-/-}$ MHC ****p<0.0001**;** **(e)** WT IsC vs WT MHC *p=0.02 , WT IsC vs $cd31^{-/-}$ IsC *p=0.05**;** **(f)** $cd31^{-/-}$ IsC vs $cd31^{-/-}$ MHC *p=0.011

(**g**) Expression of the enzyme cpt1a mRNA by WT and *cd31 -/-* EC stimulated for 4 hours with anti-MHC or control Isotype-matched antibody was measured by RT-PCR. Data are shown as mean ± SD of three replicates. (N=3 independent experiments). Two-sided Student T test, *p = 0.04174

Supplementary Figure 4

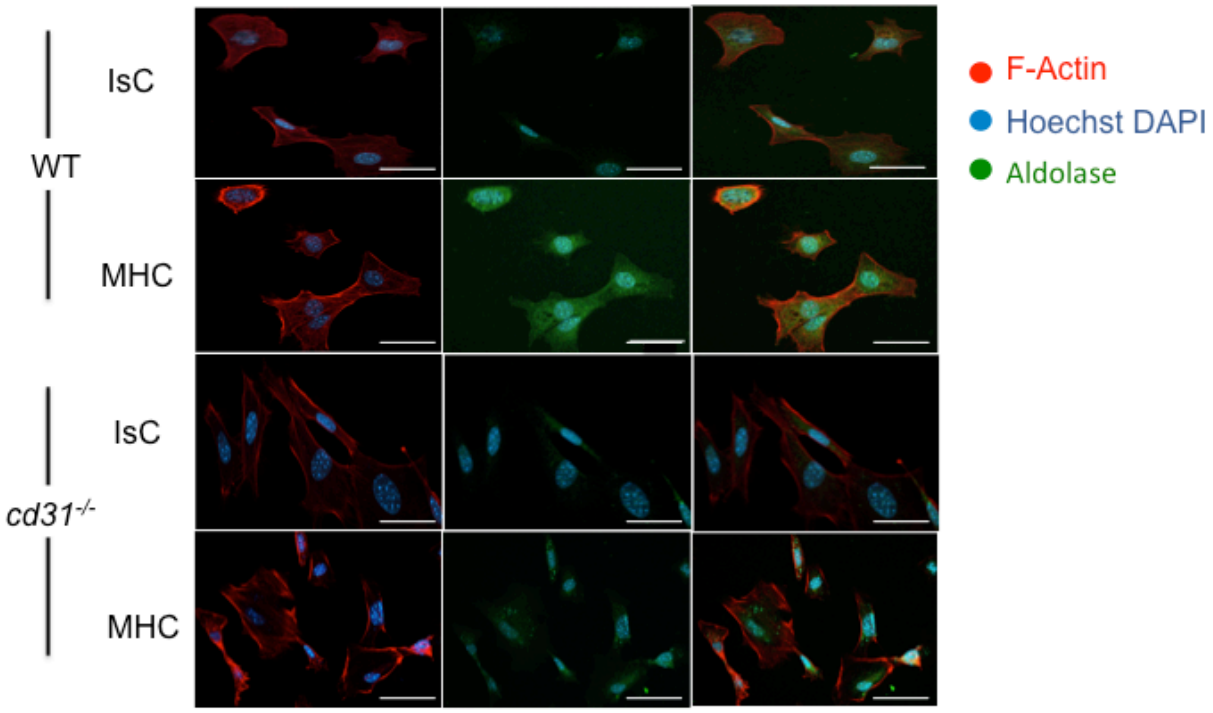

Aldolase expression and cellular localization. EC (6 × 10^4/well) previously treated with 300 U/ml IFN-γ for 48 hours (to enhance MHC molecule expression), were stimulated with 5 μg/ml anti-mouse H-2Ld/H-2Db followed by a secondary cross-linking Ab. Expression of F actin and Aldolase was assessed 30 minutes later by confocal microscopy. N=2 Scale bar, 30 μm. Magnification ×20. (N=3 independent experiments)

Supplementary Figure 5

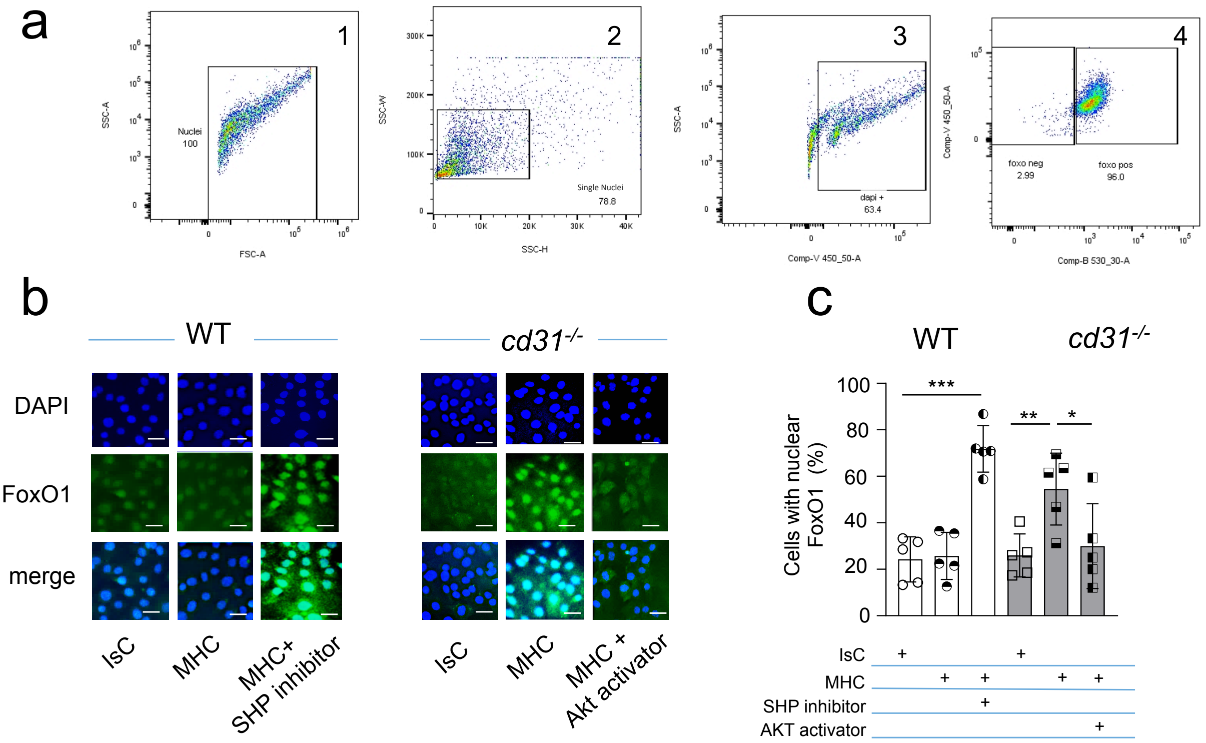

FoxO1 nuclear exclusion requires CD31-induced SHP and Akt activation.

(**a**) Gating strategy for analysis of nuclear FoxO1. Isolated nuclei were identified by single and side scatter (1) and following gating on single nuclei (2) and DAPI (3) cells and FoxO 1 expression was analyzed (4).

WT and CD31-deficient EC were stimulated by MHC antibody-ligation or treated with an Isotype-Matched Control and secondary antibody. WT EC were also exposed to the SHP1/2 inhibitor and CD31-deficient EC were pre-treated with an Akt activator (500nM) for 3 hours before stimulation. (**b**) EC were stained using rabbit anti-mouse FoxO1 (*green*) and DAPI (blue) Representative immunofluorescence images taken 2 hours after stimulation are show. Bar = 20 μm. (N=3 independent experiments)

(**c**) For quantification, 500 cells per coverslip were analyzed, and the bar graph shows the percentage of cells displaying nuclear FoxO1 localization measured in three experiments of

identical design. Data are shown as mean ± SD. N=2 independent experiments. One-way Anova with Tuckey post-hoc test. WT IsC vs WT MHC+SHP inhibitor ***p=0.0003 , *cd31*$^{-/-}$ IsC vs *cd31*$^{-/-}$ MHC **p=0.02 , *cd31*$^{-/-}$ MHC vs *cd31*$^{-/-}$ MHC+Akt activator *p=0.0114

Supplementary Figure 6

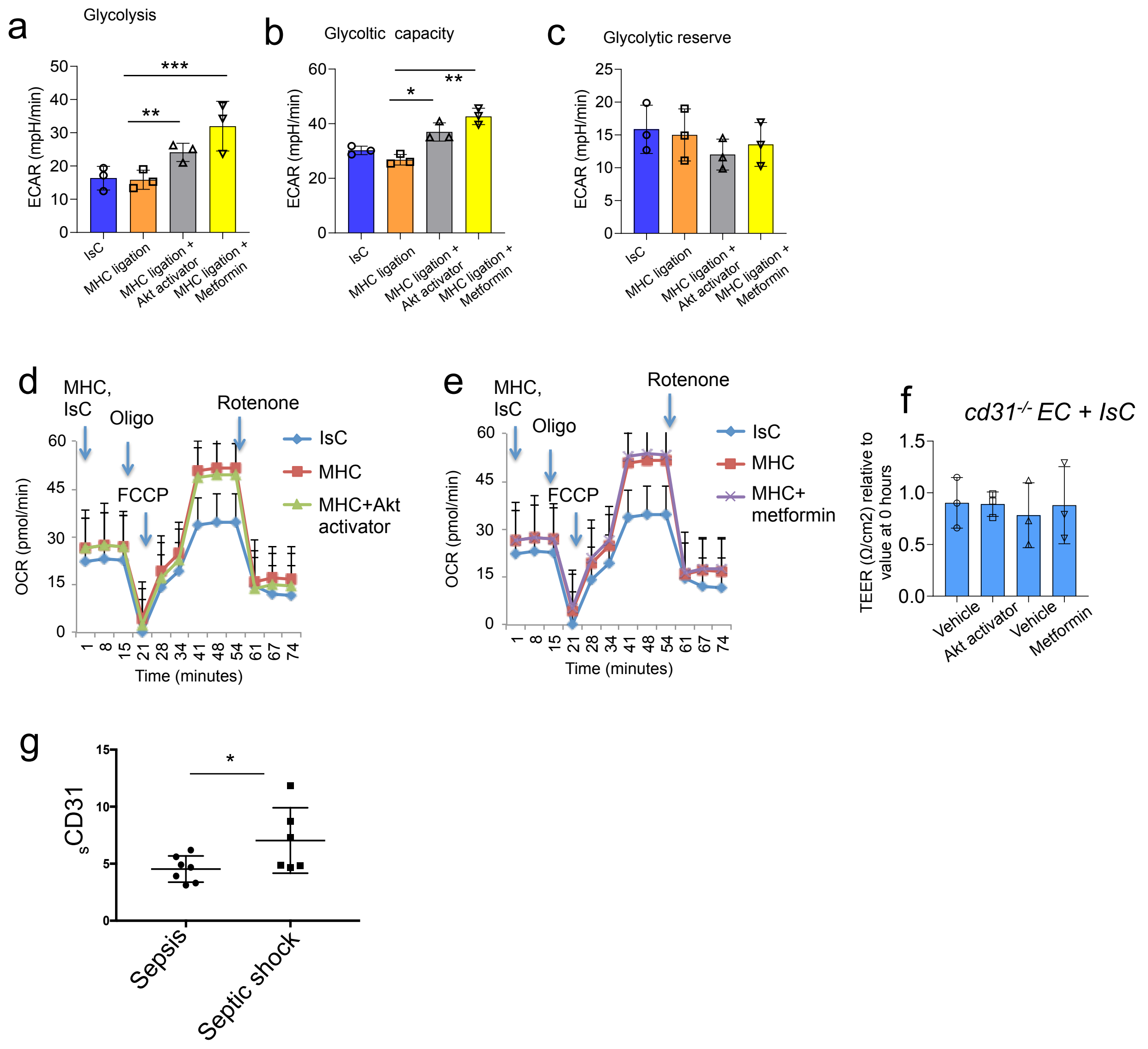

Akt and AMPK activation restore CD31-deficient EC glycolytic response to MHC stimulation. CD31-deficient EC were treated with an Akt activator (500 nM) or Metformin (5mM), for 3 hours prior measurement of the glycolytic flux. The basal and maximal glycolysis and the glycolytic reserve are shown in panels **a,** **b**, and **c**, respectively. The error bars represent SD. (N=3 independent experiments). One-way Anova with Tuckey post-hoc test. (a) MHC ligation vs MHC ligation+Akt activator **p=0.0021 , MHC ligation vs MHC ligation+Metformin ***p=0.0002; (b) MHC ligation vs MHC ligation+Akt activator *p=0.0479, MHC ligation vs MHC ligation+Metformin

**p=0.0054

The mean oxygen consumption rate (OCR) of antibody-stimulated CD31-deficient EC treated with an Akt activator or Metformin is shown in panels **d** and **e,** respectively (N=2 independent experiments). The error bars represent SD. One-way Anova with Tuckey post-hoc test.

(**f**) Quantitative analysis of TEER of CD31-deficient EC stimulated with an IsC and cross-linking antibodies and simultaneously exposed to Akt activator (500 nM) or Metformin (5mM), for 3 hours prior to measurement. Error bars represent SD. (N=2 independent experiments, n=3 biologically independent samples)

(**g**) Soluble (s)CD31 was measured in plasma from patients with sepsis (n=7 patients) or septic shock (n=7 patients) by ELISA. Error bars represent SD. Two-sided Student T test, *p=0.05

Supplementary Table 1

Characteristics of patients with sepsis

Age	Gender	Underlying condition
85	M	benign hypertension, heart disease
80	M	Pancreatic cancer
46	F	colorectal cancer
71	M	diabetes, hypertension, dyslipidemia, atrial fibrillation
47	M	NAFLD
88	M	aspiration pneumonia, acute kidney disease
64	M	Anemia, malabsorption syndrome, HBV postive

Supplementary Table 2

Characteristics of patients with septic shock.

Age	Gender	Underlying condition
83	M	diverticulitis
70	F	Pancreatic cancer in situ, peritoneal abscess, peritonitis
83	M	heart failure, chronic ischemic cardiomyopathy, thrombocytopenia
83	F	heart failure, anuria, acute kidney disease
90	F	Epilepsy, cerebrovascular disease
81	F	bladder cancer, surgical wound infection
64	M	Acute kidney disease, gallbladder stones, HBV positive
