# Supplementary material for: Preservation of microvascular barrier function requires CD31 receptor-induced metabolic reprogramming
Source: Nat Commun. 2020 Jul 17;11:3595. doi: 10.1038/s41467-020-17329-8 (PMC7367815; doi:10.1038/s41467-020-17329-8)
Supplement: Supplementary file 2 — Reporting Summary [file 41467_2020_17329_MOESM2_ESM.pdf]

nature research

Corresponding author(s): Federica M Marelli-Berg

Last updated by author(s): May 18, 2020

Reporting Summary

Nature Research wishes to improve the reproducibility of the work that we publish. This form provides structure for consistency and transparency in reporting. For further information on Nature Research policies, seeAuthors & Referees and theEditorial Policy Checklist .

Statistics

For all statistical analyses, confirm that the following items are present in the figure legend, table legend, main text, or Methods section.

n/a	Confirmed	
☐	☒	The exact sample size (*n*) for each experimental group/condition, given as a discrete number and unit of measurement
☐	☒	A statement on whether measurements were taken from distinct samples or whether the same sample was measured repeatedly
☐	☒	The statistical test(s) used AND whether they are one- or two-sided<br>*Only common tests should be described solely by name; describe more complex techniques in the Methods section.*
☒	☐	A description of all covariates tested
☒	☐	A description of any assumptions or corrections, such as tests of normality and adjustment for multiple comparisons
☒	☐	A full description of the statistical parameters including central tendency (e.g. means) or other basic estimates (e.g. regression coefficient) AND variation (e.g. standard deviation) or associated estimates of uncertainty (e.g. confidence intervals)
☒	☐	For null hypothesis testing, the test statistic (e.g. *F*, *t*, *r*) with confidence intervals, effect sizes, degrees of freedom and *P* value noted<br>*Give P values as exact values whenever suitable.*
☒	☐	For Bayesian analysis, information on the choice of priors and Markov chain Monte Carlo settings
☒	☐	For hierarchical and complex designs, identification of the appropriate level for tests and full reporting of outcomes
☒	☐	Estimates of effect sizes (e.g. Cohen's *d*, Pearson's *r*), indicating how they were calculated

Our web collection on statistics for biologists contains articles on many of the points above.

Software and code

Policy information about availability of computer code

Data collection	Images were acquired with (wide-field microscopy) and with Zeiss LSM800 (confocal microscopy)<br>Flow cytometry were acquired with LRS1 Fortessa (BD Bioscience, equipped with for lasers 488;nm blue laser, 461 nm yellow-green laser, 641 nm red laser, 405 nm violet laser).<br>Real-time fluxometry data were taken with a Seahorse XFe96 Analyzer<br>Quantitative PCR data were collected with CFX Connect Real-Time PCR Detection System
Data analysis	Images were analyzed with ImageJ Software v 1.37c<br>Flow cytometry data were analysed with FlowJo Software (v10.5.3)<br>Real Time Fluxometry data were analyzed with Wave v2.4.1 Software<br>Quantitative PCR data were analyzed withLightCycler 480 Software<br>12-bit images were generated using Axiovision Software v 4.8<br>Sequences of qPCR Primers were designed by using PrimerPlus v3<br>Statisticcal Analysis was performed with GraphPad Prism (v8.0.1)

For manuscripts utilizing custom algorithms or software that are central to the research but not yet described in published literature, software must be made available to editors/reviewers. We strongly encourage code deposition in a community repository (e.g. GitHub). See the Nature Research guidelines for submitting code & software for further information.

Data

Policy information about availability of data

All manuscripts must include a data availability statement. This statement should provide the following information, where applicable:

- Accession codes, unique identifiers, or web links for publicly available datasets
- A list of figures that have associated raw data
- A description of any restrictions on data availability

All data supporting the results presented herein are available from the corresponding authors upon reasonable request. The source data for all the graphs and un-cropped gels and blots in the main Figures and Supplementary Information are provided as a Source Data file.are provided as a Source Data file.

Field-specific reporting

Please select the one below that is the best fit for your research. If you are not sure, read the appropriate sections before making your selection.

☒ Life sciences ☐ Behavioural & social sciences ☐ Ecological, evolutionary & environmental sciences

For a reference copy of the document with all sections, see nature.com/documents/nr-reporting-summary-flat.pdf

Life sciences study design

All studies must disclose on these points even when the disclosure is negative.

Sample size	Sample size was chosen based on previous experience that such size would be sufficient to provide a power of 80% at the 5% significance level
Data exclusions	No data points were excluded from the analysis
Replication	All analyses were done using at least 3 independent biological replicates/mice, and repeated at lease twice in separate experiments at different time points. All attempt at replication were successful
Randomization	Randomization in our study was not necessary as the mice are genetically identical and inbread. Patients required diagnosis before testing and this is not a clinical trial.
Blinding	Blinding was not used in this study as data were mostly obtained by automatic software measurement and most experiments were carried out by a single worker

Reporting for specific materials, systems and methods

We require information from authors about some types of materials, experimental systems and methods used in many studies. Here, indicate whether each material, system or method listed is relevant to your study. If you are not sure if a list item applies to your research, read the appropriate section before selecting a response.

Materials & experimental systems

n/a	Involved in the study	
☐	☒	Antibodies
☒	☐	Eukaryotic cell lines
☒	☐	Palaeontology
☐	☒	Animals and other organisms
☐	☒	Human research participants
☐	☒	Clinical data

Methods

n/a	Involved in the study	
☒	☐	ChIP-seq
☐	☒	Flow cytometry
☒	☐	MRI-based neuroimaging

Antibodies

Antibodies used

Rabbit-anti-mouse-Phospho-Akt (Ser473) Antibody (Cell signalling, AB329825), Rabbit-anti-mouse-Akt (pan) (C67E7) Antibody (Cell signaling AB915783), Rabbit-anti-mouse- P-ERK1/2 Antibody (Cell signalling, AB331646), Rabbit-anti-mouse ERK1/2 Antibody (Santa Cruz, Sc292838), Mouse-anti-mouse P-tyrosine (PY20) Antibody (Santa Cruz, Sc 508), Rabbit-anti-mouse SHP2 Antibody (Abcam ab131541), Rabbit-anti-mouse Ubiquitin Antibody (Abcam ab7780), Goat anti-actin-I19 Antibody (Santa Cruz Biotechnology, Sc1616), Rabbit-anti-mouse PFKFB3 (D7H4Q) Antibody (Cell signalling, 13123), Rabbit-anti-mouse Enolase-2 (D20H2) Antibody (Cell signaling, AB11178392), Rabbit-anti-mouse Enolase-1 Antibody (Cell signalling, AB2246524), Rabbit-anti-mouse Aldolase AAntibody (Cell signalling, AB2226674), Rabbit-anti-mouse PGAM1 Antibody (Cell signalling, 12098), Anti-rabbit IgG, HRP-linked Antibody (Cell signalling, AB2099233), Rabbit-anti-mouse rac1/cdc42 Antibody (Cell signalling, AB10612265), Rabbit-anti-mouse PE-conjugated anti- CD31 Antibody (Thermofisher AB465631), Rat IgG2a K Isotype Control Antibody (Thermofisher AB470104), Rabbit-anti-mouse p-β-catenin (phospho Y654) Antibody (Abcam ab59430), Rabbit-anti-

mouse VE-cadherin (phospho Y685) Antibody (Abcam AB10971838), Rabbit-anti-mouse VE-cadherin Antibody (Abcam ab205336). Rabbit-anti-mouse β-Catenin Antibody (Abcam AB11127855) ,Rabbit-anti-mouse Anti-PFKFB3 Antibody (Abcam AB181861), Rabbit-anti-mouse Anti-beta Actin Antibody (Abcam AB16039), Rabbit-anti-mouse c-Myc Antibody (Abcam AB10858578), Alexa Fluor® 555 Goat-anti-mouse Ig Antibody (Life Technology AB2563179), and FITC Donkey anti-rabbit IgG (minimal x-reactivity) Antibody (Life Technology AB893531), Rabbit-anti-mouse FoxO1 Antibody (Abcam AB2106495), Mouse-anti-mouse anti-Glut1-PE Antibody (Novusbio, NB110-39113PE), Rabbit-anti-mouse anti-Erk1/2 (H72) Antibody (Santa Cruz , SC292838), tetramethyl rhodamine B isothiocyanate-phalloidin (Sigma , P1951). Rabbit-anti-mouse Glut1 Antibody AF647 (Novus Bio, NB110-39113AF647), Rabbit monoclonal to Aldolase antibody (Abcam, ab169544), Phalloidin staining Alexa Fluor™ 568 (Invitrogen™, A12380)

Mouse-anti-mouse H-2Ld/H-2Db (MHC class I) Antibody (Biolegend, 114502), Rat–anti-mouse IgG, (Biolegend , 553445), (Blocking) rat anti-mouse CD31 clone 390 (eBioscience , AB10060377), (Stimulate) rabbit anti-mouse CD31 (Abcam, ab2836), Goat–anti-rabbit Ig Antibody, (Agrisera , AS10 665), (cross-linkage) Mouse-anti-mouse ICAM-1 (Abcam, ab2213) were used to stimulate MHC molecules on the endothelium.

Other antibodies used in this study mouse–anti-mouse H-2Ld/H-2Db (BD Biosciences 14-5999-85), goat–anti-mouse IgG (BioLegend 405301), rat–anti-mouse CD54 (eBioscience 14-0549-82, goat anti-rat Ig (Biolegend 400202), Glut 1 AF647 antibody (Novus Biological, NB110-39113) , Aldolase 488 (Novus Biological, 42620AF488), Phalloidin 586 antibodies (ThermoFisher Scientific A12380)

Validation	All antibodies have been validated by the manufacturers using All antibodies have been validated by the manufacturers using Cell treatment Validation: detecting downstream events following the treatment, Knockdown Validation via the expression using RNAi to knock down the gene of interest, Knockout Validation by using CRISPR-Cas9 cell models, Neutralisation Validation by functional blockage of protein activity by antibody binding, Indipendent antibody verification: measurement of target expression is performed using two differentially raised antibodies recognizing the same protein target.

Animals and other organisms

Policy information about studies involving animals; ARRIVE guidelines recommended for reporting animal research

Laboratory animals	C57Bl/6 mice purchased from Charles River. Male and female mice. Mice (cd31 KO and littermates) were bred in house and used at 6-10 weeks of age. Housed in SPF conditions.
Wild animals	The study did not involve wild animals
Field-collected samples	The study did not involve samples collected from the field
Ethics oversight	All in-vivo experiments were conducted with strict adherence to the Home Office guidelines (PPL P71E91C8E) following approval by the Queen Mary University of London Ethics committee. The number of animals required to obtain statistical significance was estimated based on similar studies previously performed. Animals were not randomized, and no blinding was done.

Note that full information on the approval of the study protocol must also be provided in the manuscript.

Human research participants

Policy information about studies involving human research participants

Population characteristics	*Describe the covariate-relevant population characteristics of the human research participants (e.g. age, gender, genotypic information, past and current diagnosis and treatment categories). If you filled out the behavioural & social sciences study design questions and have nothing to add here, write "See above."*
Recruitment	*Describe how participants were recruited. Outline any potential self-selection bias or other biases that may be present and how these are likely to impact results.*
Ethics oversight	*Identify the organization(s) that approved the study protocol.*

Note that full information on the approval of the study protocol must also be provided in the manuscript.

Clinical data

Policy information about clinical studies

All manuscripts should comply with the ICMJEguidelines for publication of clinical research and a completedCONSORT checklist must be included with all submissions.

Clinical trial registration	*Provide the trial registration number from ClinicalTrials.gov or an equivalent agency.*
Study protocol	*Note where the full trial protocol can be accessed OR if not available, explain why.*
Data collection	*Describe the settings and locales of data collection, noting the time periods of recruitment and data collection.*
Outcomes	*Describe how you pre-defined primary and secondary outcome measures and how you assessed these measures.*

Flow Cytometry

Plots

Confirm that:

- [x] The axis labels state the marker and fluorochrome used (e.g. CD4-FITC).
- [x] The axis scales are clearly visible. Include numbers along axes only for bottom left plot of group (a 'group' is an analysis of identical markers).
- [x] All plots are contour plots with outliers or pseudocolor plots.
- [x] A numerical value for number of cells or percentage (with statistics) is provided.

Methodology

Sample preparation	EC were detached using Trypsin-EDTA, washed in PBS 1% FCS and kept on ice until staining. Surface stsining was performed with the indicated antibodies for 20 minutes on ice. Following staining cells were washed twice in FACS buffer.For intracellular staining, commercially available buffers were used. Acquisition was performed in FACS buffer. For 6-NBDG uptake assay, cells were treated as indicated in the material and methods section, and acquired in FACS buffer.
Instrument	BD LSR Fortessa and BD LSRII (analysis)
Software	FlowJo (10.5.3)
Cell population abundance	Samples consisted of at least 2 x10^6 cells. The cell population was 99% pure.
Gating strategy	The gating strategy is provided for all flow cytometry anlalyses in the relevant Supplementary figures. Cells were similarly gated:FSC-A/SSC-A, singlets (FSC-A/FSC-H) and a live/dead marker as specified in the Materials and Methods section.

- [x] Tick this box to confirm that a figure exemplifying the gating strategy is provided in the Supplementary Information.
